# Incorporating brain-inspired mechanisms for multimodal learning in artificial intelligence

**DOI:** 10.1126/sciadv.ady8751

**Published:** 2026-06-05

**Authors:** Xiang He, Dongcheng Zhao, Yang Li, Qingqun Kong, Xin Yang, Yi Zeng

**Affiliations:** ^1^ Brain-inspired Cognitive AI Lab, Institute of Automation, Chinese Academy of Sciences, Beijing, China.; ^2^ Center for Long-term AI, Beijing, China.; ^3^ CAS Key Laboratory of Molecular Imaging, Institute of Automation, Chinese Academy of Sciences, Beijing, China.; ^4^ Key Laboratory of Brain Cognition and Brain-inspired Intelligence Technology, Chinese Academy of Sciences, Shanghai, China.

## Abstract

Multimodal learning enhances the perceptual ability of intelligent systems by integrating information across sensory modalities. However, most artificial intelligence approaches still rely on static fusion schemes and do not account for the dynamic nature of multisensory integration observed in the brain. In biological systems, the principle of inverse effectiveness states that weaker unimodal cues contribute more strongly to multisensory integration, whereas strong cues reduce the relative benefit of fusion. Motivated by this mechanism, we investigate the relationship between multimodal outputs and modality-specific information and introduce an inverse effectiveness–driven multimodal fusion (IEMF) strategy. Integrating IEMF into neural networks yields more adaptive fusion behavior, improved accuracy, and substantial gains in computational efficiency. We validate the generality of IEMF across audiovisual perception, vision-language understanding, and trimodal sentiment analysis, demonstrating consistent improvements over state-of-the-art baselines in classification, continual learning, and question answering. The approach also transfers across architectures, including both artificial neural networks and spiking neural networks.

## Teaser

Biologically inspired inverse effectiveness enables more adaptive and efficient multimodal learning across diverse sensory tasks.

## INTRODUCTION

**Fig. 1.**
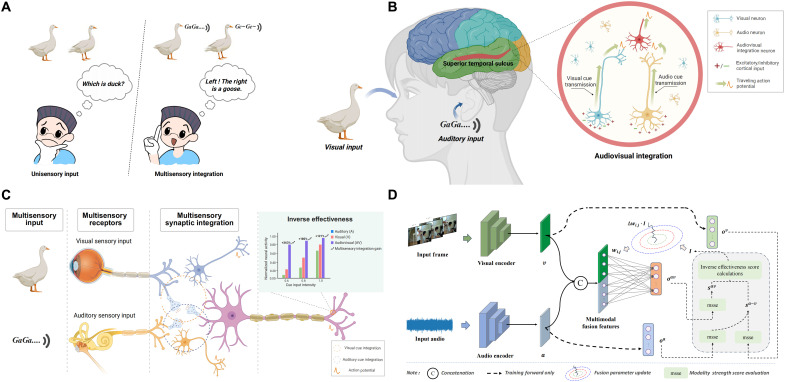
Illustration of multisensory integration and the role of inverse effectiveness in IEMF. (**A**) Comparison between unimodal sensory input and multisensory integration: Integrating visual and auditory cues reduces ambiguity and uncertainty compared to relying on a single modality. (**B**) Neural basis of audiovisual integration in the human brain, focusing on the superior temporal sulcus (STS) where visual and auditory inputs converge onto multisensory neurons. (**C**) Biological principle of inverse effectiveness: Multisensory integration is strengthened when unimodal signals are weak. Visual and auditory stimuli are processed through distinct sensory pathways and converge at multisensory synapses. The inset illustrates the inverse relationship between unimodal strength and integrative gain. (**D**) The proposed IEMF strategy inspired by biological multisensory fusion mechanisms. Visual and auditory inputs are processed by respective encoders, fused via a dynamic fusion module regulated by inverse effectiveness principles, and evaluated using modality strength score estimation. The fusion module weights are dynamically adjusted according to the computed scores. [Illustrations in (A) created by X.W. and adapted using BioRender. (A) to (C) were created in BioRender. X.H. (2026). (A) https://biorender.com/qhryo4j; (B) https://biorender.com/2cuqu7c; (C) https://biorender.com/tqqcykt.]

In natural environments, we typically need to process cues from multiple senses simultaneously to comprehensively construct an understanding of the same concept. For example, understanding the concept of “beach” involves not only visual information (yellow sand and blue sea) but also relies on auditory (sound of waves) and tactile (texture of sand) sensory information. Compared to unimodal information, multimodal information provides richer and more comprehensive representational capacity (*1*, *2*). As shown in Fig. 1A, multimodal integration not only enhances information expressiveness but also effectively reduces uncertainty in single-modal information. This mechanism of multimodal information integration is not only the foundation of biological perception but has also become one of the core challenges in multimodal learning in artificial intelligence. As information environments become increasingly complex, traditional unimodal learning methods struggle to handle complex and dynamic real-world scenarios. Consequently, neural networks have incorporated multimodal information processing strategies to achieve more robust and efficient information representation. Multimodal neural networks are widely applied in tasks such as multimodal fusion (*3*–*7*), multimodal emotion recognition (*8*, *9*), and audiovisual speech recognition (*4*, *10*–*12*). Current mainstream multimodal paradigms, such as contrastive vision-language (VL) pretraining (*13*–*15*) and large-scale transformer-based fusion architectures (*16*–*18*), have achieved remarkable performance by leveraging massive datasets and attention mechanisms. However, existing engineering paradigms typically use static or attention-based weighting strategies (*4*, *19*, *20*), which often struggle to compensate as flexibly as biological systems when facing high noise or drastically fluctuating modal quality in complex real-world scenarios. Therefore, how to design a plug-and-play mechanism that enables artificial neural networks (ANNs) to dynamically adjust learning strategies based on modal reliability, akin to the biological brain, remains a critical unresolved issue. Beyond architectural advances, a complementary line of work explicitly balances modality contributions through adaptive attention, confidence-aware fusion, and uncertainty-based weighting. Representative methods include homoscedastic-uncertainty weighting for multitask learning (*21*), confidence-weighted multimodal fusion (*22*), and gradient modulation–based approaches such as on-the-fly balancing (*4*), and curriculum-based multimodal training (*23*). These studies fundamentally validate the necessity of modulating optimization trajectories on the fly, establishing a computational imperative that moves beyond static weights and paves the way for integrating more sophisticated, biologically grounded adaptive mechanisms. Nevertheless, brain-inspired algorithms remain in the early stage, and many biological characteristics and mechanisms have not yet been fully used, offering enormous potential and emerging challenges for the further development of neural network models.

Neurobiological research indicates that vision and audition are the two primary pathways through which humans acquire external information, and their integration notably enhances perceptual benefits (*24*, *25*). Given that audiovisual integration is one of the most thoroughly documented forms of cross-modal interaction in neuroscience, we focus on vision-audition integration as the primary source of conceptual intuition from which our inverse effectiveness mechanism is derived and subsequently extend it to other multimodal settings. For visual and auditory information from a common source, the brain has specialized regions responsible for both unimodal information processing and multisensory integration (*26*). After visual and auditory information are received through their respective receptors, features are hierarchically extracted through visual and auditory pathways before being transmitted to multisensory integration brain regions. Relevant studies show that audiovisual information integration occurring in the cerebral cortex is closely associated with regions such as the superior temporal sulcus (*27*–*32*), posterior parietal cortex (*33*, *34*), and prefrontal cortex (*35*, *36*). Figure 1B illustrates the convergence and integration process of cosourced audiovisual signals in the superior temporal sulcus as an example. In this multisensory integration brain region, visual and auditory cues are transmitted through different neural pathways, ultimately converging onto common multisensory integration neurons, facilitating cross-modal information integration and perceptual decision-making.

Brain audiovisual information integration exhibits many interesting mechanisms, with inverse effectiveness being particularly noteworthy. Calvert *et al.* (*27*) found that by separately presenting audiovisual combined speech signals and their unimodal information and conducting cross-modal comparisons, the left superior temporal sulcus demonstrated the most significant cross-modal integration benefits under conditions where unimodal signals were weakest. The inverse effectiveness mechanism indicates that during multisensory information integration, when unimodal cues are weaker, the effect of multisensory integration is relatively stronger; conversely, when individual modal cues are stronger, the effect of modal fusion is relatively diminished, although multisensory integration responses still exceed the activation response of either single modality (*37*, *38*). Figure 1C illustrates this phenomenon, where inverse effectiveness reflects higher sensitivity to weaker modalities in multimodal integration brain regions, typically manifested as enhanced information integration. This mechanism enables biological systems to enhance perceptual accuracy and stability by strengthening multimodal integration when the quality of a single modality is poor.

Inspired by the biological principle of inverse effectiveness, our work reconsiders how multimodal fusion should adapt to variations in unimodal input quality, particularly under dynamic and complex environments. Most existing multimodal fusion methods focus on maximizing information interaction between modalities yet often overlook the dynamic relationship between the fused output and the respective contributions of each modality. This limitation stems from existing methods typically presetting modal interactions as static fixed patterns, failing to fully consider that different modalities’ information contributions should flexibly adjust as environmental conditions change. Distinct from existing engineering approaches that aim to design specific fusion architectures, we propose a general, architecture-agnostic gradient modulation strategy. Take audiovisual perception as an example: When environmental noise substantially degrades the quality of auditory input, traditional fusion strategies typically retain fixed fusion weights and cannot adaptively modify the cooperative interaction between modalities in accordance with signal degradation, thereby constraining the overall perceptual performance of the system. The phenomenon of inverse effectiveness inspires the insight that an efficient multimodal integration mechanism should actively enhance the responsiveness of the fusion module when the quality of a single modality degrades so that the system can obtain more compensatory information from other modalities. On the basis of this insight, we propose that fusion strength should dynamically respond to modality-specific quality fluctuations, that is, the learning rate of the fusion module should be adaptively modulated according to the reliability of unimodal signals, thereby enabling more robust and flexible multimodal perception in complex and evolving environments.

Drawing conceptual intuition from the aforementioned neural mechanisms of multimodal fusion and biological inspiration, this paper adopts deep neural networks as the foundational framework for multimodal perceptual learning, focusing on investigating the dynamic interaction and cooperative integration mechanisms among modalities. We characterize the neuroscience findings as a source of conceptual intuition, serving as a guiding heuristic. Our goal is to translate the functional essence of inverse effectiveness into a robust, architecture-agnostic training strategy, thereby bridging biological efficiency with artificial optimization. To this end, we propose an inverse effectiveness–driven multimodal fusion (IEMF) strategy to enable a more fine-grained fusion mechanism (Fig. 1D). Although IEMF originates from audiovisual neural mechanisms, it is designed as a universal training-time optimization mechanism that can be applied in a plug-and-play manner to various modality combinations and architectures. IEMF operates solely during training and does not modify the inference-time architecture or computation, thereby ensuring that it introduces no additional runtime overhead. By quantifying the relationship between the strength of unimodal inputs and the modality strength of multimodal fusion outputs, we adaptively modulate the update rate of the fusion module’s weights. Specifically, we introduce an inverse effectiveness coefficient into the backpropagation process such that the fusion module accelerates its parameter updates in response to weak unimodal signals to enhance fusion strength, while suppressing updates when unimodal signals are strong, thereby reducing overreliance on fusion. This design realizes a biologically inspired principle of “weak modality, strong fusion,” ensuring that the integration process neither overdepends on a single sensory pathway nor overlooks potentially informative sources. Consequently, the proposed method not only enhances overall perceptual accuracy and robustness but also achieves remarkable computational efficiency. Specifically, it reduces training costs by up to 50% while maintaining exceptional performance.

Overall, our contributions can be categorized into the following three points:

1.We introduce the inverse effectiveness mechanism into multimodal fusion in deep neural networks, proposing an IEMF strategy. This strategy adjusts the parameter update intensity of fusion modules in real time, enabling the model to enhance its ability to extract information from other modalities when a single modality signal is weak, thereby improving information compensation effects and fusion efficiency.

2.We conduct systematic empirical studies on multiple standard datasets and tasks, including representative scenarios such as audiovisual speech recognition, audiovisual continual learning, and audiovisual question answering (AVQA), and further extend our evaluation beyond audiovisual integration to VL understanding and text-audiovisual dialogue understanding. Experimental results show that our proposed method exhibits stronger perceptual capabilities under various complex conditions. Particularly worth emphasizing is that, as a mechanism, IEMF can seamlessly integrate with various existing state-of-the-art methods and further enhance their performance.

3.We validate the universality of IEMF across distinct information-processing paradigms, ranging from ANNs to spiking neural networks (SNNs). Furthermore, we demonstrate that IEMF acts as an architecture-agnostic gradient modulation mechanism that is naturally compatible with diverse backbone topologies, including both Convolutional Neural Networks (CNNs) and transformers. Experimental results demonstrate that IEMF has good generality and can be effectively integrated with both types of networks.

## RESULTS

### Performance across diverse multimodal fusion scenarios

#### Audiovisual classification

**Fig. 2.**
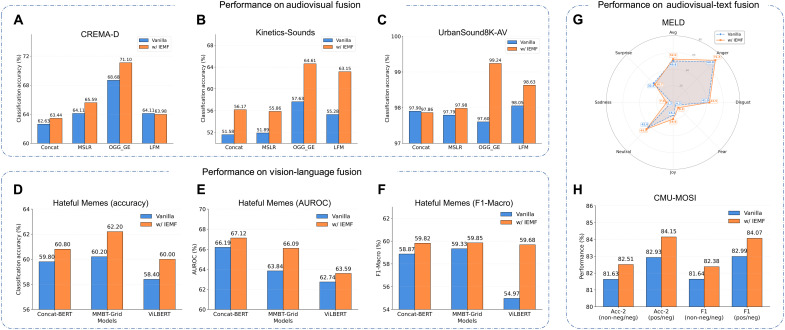
IEMF performance across different modality combinations. Audiovisual fusion. Performance comparison on three standard benchmarks [CREMA-D (**A**), Kinetics-Sounds (**B**), and UrbanSound8K-AV (**C**)], showing consistent improvements of IEMF (orange) over the vanilla baseline (blue) across multiple fusion strategies. VL fusion. Evaluation on the Hateful Memes dataset using three representative models (Concat-BERT, MMBT-Grid, and VILBERT). IEMF boosts performance across all metrics: Accuracy (**D**), AUROC (**E**), and F1-Macro (**F**). Audiovisual-text fusion. (**G**) Radar chart on the MELD dataset illustrating the per-emotion recognition improvements (e.g., sadness, anger). (**H**) Performance on the CMU-MOSI dataset, where IEMF achieves higher binary accuracy (Acc-2) and F1 scores compared to the vanilla method.

We systematically validated the effectiveness of the IEMF mechanism in audiovisual classification tasks. We evaluated performance differences between baseline models and IEMF-enhanced models across three representative datasets: CREMA-D (*39*), Kinetics-Sounds (*40*), and UrbanSound8K-AV (*41*), using four mainstream fusion strategies: concatenation fusion (Concat), modality-specific learning rates (MSLR) (*19*), on-the-fly gradient modulation with generalization enhancement (OGM_GE) (*4*), and learning facilitator for modality (LFM) gap (*20*). As shown in Fig. 2 (A to C), IEMF demonstrates consistent performance improvements across all fusion schemes and datasets. Specifically, taking IEMF’s enhancement to the MSLR method across datasets as an example, on the CREMA-D dataset, the baseline model achieved 64.11% accuracy using MSLR, which improved to 65.59% after introducing IEMF, yielding a 1.48% gain. On the more challenging Kinetics-Sounds dataset, baseline accuracy was 51.89%, while the IEMF-enhanced model reached 55.86%, a 3.97% improvement. Even on the UrbanSound8K-AV dataset where the baseline model already achieved high accuracy of 97.79%, IEMF further improved it to 97.98%. Although limited in magnitude, this improvement remains practically significant given the already high performance level.

Looking at overall trends, IEMF consistently improves performance across datasets and fusion strategies, confirming its effectiveness in enhancing multimodal fusion efficiency. Unlike traditional fusion methods, IEMF dynamically adjusts modal fusion module weights based on each modality’s relative strength. When information in one modality (e.g., audio) decreases because of noise or distortion, IEMF promotes greater information compensation from the fusion module, improving overall perceptual accuracy and model robustness. This dynamic adaptive mechanism notably enhances model robustness when facing input quality fluctuations and environmental uncertainties.

#### VL fusion

To demonstrate that IEMF is not limited to audiovisual tasks, we first extended our evaluation to VL scenarios using the Hateful Memes dataset (*42*). We used three representative architectures: Concat BERT (*42*), MMBT-Grid (*43*), and ViLBERT (*44*). As shown in Fig. 2 (D to F), IEMF consistently boosted performance across accuracy, area under the receiver operating characteristic curve (AUROC), and F1-Macro metrics for all architectures. Specifically, for the Concat BERT model, IEMF improved the AUROC from 66.19 to 67.12%. On the MMBT-Grid model, the classification accuracy increased from 60.20 to 62.20%, achieving a gain of 2.0%. Notably, for the ViLBERT architecture, IEMF improved the F1-Macro score from 54.97 to 59.86%, demonstrating its capability to optimize fusion even in complex transformer-based models.

#### Trimodal fusion

Furthermore, we validated the efficacy of IEMF in a trimodal setting (text-audiovisual), capturing complex interactions among three modalities. We conducted experiments on the MELD (*45*) (emotion recognition) and CMU-MOSI (*46*) (sentiment analysis) datasets, as illustrated in Fig. 2 (G and H). On the MELD dataset, IEMF achieved a superior weighted-F1 score of 52.03%, compared to 49.77% for the vanilla baseline. Significant improvements were observed in recognizing challenging emotions, such as “Fear” (improving from 4.26 to 8.82%) and “Joy” (improving from 15.25 to 19.88%). Similarly, on the CMU-MOSI benchmark, IEMF consistently outperformed the baseline on both classification tasks. For instance, the binary accuracy (Acc-2) for the positive/negative task increased from 82.93 to 84.15%, and the F1 score for the nonnegative/negative task improved from 81.64 to 82.38%. These results verify IEMF’s effectiveness in handling multimodal integration and improving sentiment analysis performance.

### Generalizability across continual learning and QA tasks

#### IEMF improved the model performance on audiovisual continual learning

**Fig. 3.**
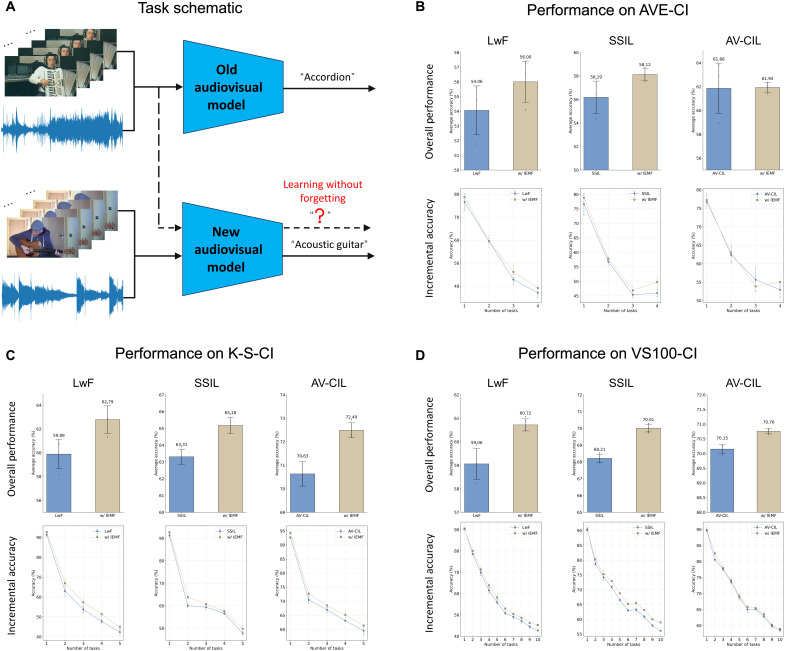
IEMF boosts audio visual continual learning. (**A**) Task schematic. A single audiovisual model is incrementally updated as new classes arrive; the goal is to absorb the new knowledge while preserving performance on previously learned classes, achieving ``learning without forgetting.” (**B**) Results on AVE-CI. (**C**) Results on K-S-CI. (**D**) Results on VS100-CI. For three representative class incremental learning baselines, LwF, SSIL and AV-CIL, we compare the vanilla method (blue) with the method augmented by IEMF (khaki). Each subpanel is split into top and bottom: The top bar chart reports the overall performance (mean accuracy across all tasks; error bars denote 1 SD), while the bottom line plot traces the incremental accuracy after each successive task. Across all datasets and baselines, IEMF consistently increases mean accuracy and yields a flatter accuracy-decay curve, indicating the better knowledge transfer.

To evaluate the effectiveness of IEMF in more challenging scenarios, we further examined its performance in audiovisual continual learning tasks. In such tasks, models need to learn new categories continuously while preserving recognition capabilities for previously learned categories as much as possible, thereby avoiding catastrophic forgetting, as shown in Fig. 3A. We selected three representative class-incremental learning baseline methods for comparison: LwF (*47*), SSIL (*48*), and AV-CIL (*49*), and evaluated them on three audiovisual continual learning datasets: AVE-CI, K-S-CI, and VS100-CI (*49*). The experimental results are shown in Fig. 3 (B to D).

After introducing the IEMF method, the models achieved stable accuracy improvements across all datasets. On AVE-CI, LwF increased from 54.06 to 56.00% (+1.94%), SSIL improved from 56.19 to 58.12% (+1.93%), and AV-CIL slightly increased from 61.86 to 61.94% (+0.08%). In K-S-CI, which features more crossmodal noise, LwF rose from 59.89 to 62.79% (+2.90%), SSIL improved from 63.31 to 65.18% (+1.87%), and AV-CIL increased from 70.63 to 72.49% (+1.86%). For the largest-scale dataset VS100-CI, LwF improved from 59.06 to 60.72% (+1.66%), SSIL from 68.21 to 70.01% (+1.80%), and AV-CIL from 70.15 to 70.76% (+0.61%). All nine comparisons showed positive gains, with an average improvement of approximately 1.63 percentage points, highlighting the consistent effectiveness of IEMF.

In Fig. 3 (B to D), the line graphs in the bottom row of each subfigure show the average accuracy changes after each continuous task. Notably, compared to baseline models, the accuracy decline curves of IEMF models are more gradual. This indicates that IEMF enhances the model’s ability to retain existing knowledge during cross-task knowledge transfer while effectively integrating information about new categories, thereby mitigating catastrophic forgetting.

IEMF does not explicitly introduce learnable parameters bound to specific tasks but rather adaptively regulates the update dynamics of the fusion module based on changes in the effectiveness of unimodal and multimodal signals, thereby implicitly adapting to modal variations across different task stages during continuous learning. Through the training process guided by the inverse effectiveness, the model can naturally adapt to changes in modal reliability during weight updates, thus reducing overreliance on a single modality when perceptual conditions fluctuate. Benefiting from this adaptive optimization strategy, IEMF not only improves the average accuracy across all tasks but also maintains a smoother performance degradation curve, preserving high overall performance and cross-task knowledge coherence even as new tasks are continuously introduced.

#### IEMF improved the model performance on AVQA

**Fig. 4.**
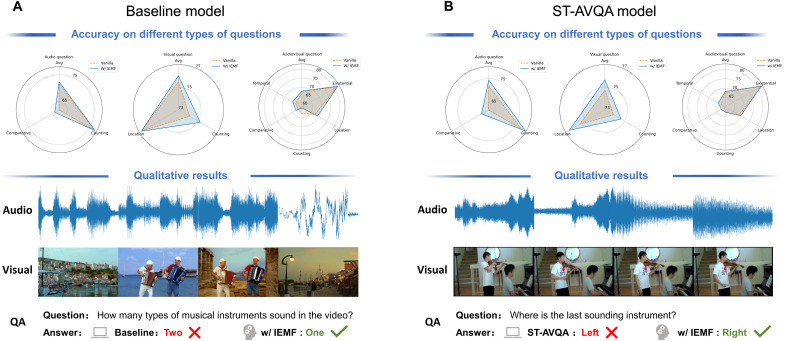
Quantitative and qualitative impact of IEMF on AVQA task. (**A**) Baseline model. The three radar charts (top) report accuracy on audio-only, visual-only, and audiovisual questions, respectively. Orange, vanilla, blue, w/ IEMF. The bottom row shows a representative sample, waveform, video frames, and question/answer, where the vanilla fusion miscounts the instruments (“Two”), whereas IEMF answers correctly (“One”). (**B**) ST-AVQA model. The same layout as (A) but using the stronger ST-AVQA model. IEMF again enlarges the radar area for every question type and corrects the localization query in the illustrated example (vanilla: “Left”; w/ IEMF: “Right”). Across both models, the blue polygons consistently enclose the orange ones, confirming that the IEMF mechanism improves all question categories while providing intuitive per-sample gains.

We further evaluated the effectiveness of IEMF in AVQA tasks. In this task, models must answer text questions based on synchronized audio and video inputs, demanding higher capabilities for deep integration of multimodal information. As shown in Fig. 4, the radar charts in Fig. 4 (A and B) compare the classification accuracy of baseline models versus models with IEMF, and ST-AVQA (*50*) models versus models with IEMF, across different question types (audio-only questions, visual-only questions, and audiovisual combined questions).

Comparing the radar charts of original models and models with IEMF, we can observe that IEMF improved answer accuracy across all question types. Taking the ST-AVQA model and its IEMF-enhanced version as an example (Fig. 4B), for audio-only questions, the original ST-AVQA model achieved an average accuracy of 71.90%, while the IEMF model improved to 74.49%, an increase of 2.59%. Similarly, for visual-only questions, the baseline accuracy was 74.74%, while the IEMF-enhanced model reached 75.65%, an improvement of 0.91%. For audiovisual questions, the vanilla model’s average accuracy was 67.61%, while the IEMF model achieved 68.33%, an improvement of 0.72%. To verify IEMF’s performance on fine-grained questions, we specifically analyzed its effectiveness in tasks requiring precise localization classification. As shown in Fig. 4B, the original ST-AVQA model incorrectly predicted “left” side when answering “the position of the last sounding instrument,” while the model with IEMF correctly located it as “right” side. This demonstrates that IEMF-enhanced models have stronger fine-grained discrimination capabilities in complex cross-modal reasoning tasks, improving the integration efficiency of multimodal cues.

This improvement further validates IEMF’s crucial advantages in handling noisy interference or incomplete input information scenarios. By dynamically adjusting the update rate of the fusion module during training based on the strength of unimodal and multimodal fusion signals, IEMF guides the model to learn strategies that can more robustly integrate different modal information when modality strengths are uneven or information is contradictory. In contrast, models without IEMF are more prone to judgment biases when facing modal conflicts or input uncertainties, leading to incorrect answers or overall performance degradation. Overall, these results highlight IEMF’s important role in enhancing multimodal understanding and reasoning.

### Generalizability across network architectures and computational paradigms

A key advantage of IEMF is its architectural universality. As demonstrated in the previous sections, IEMF effectively boosts performance across a wide range of architectures: from the sequential modeling of recurrent networks (e.g., LSTMs) and local feature extraction of convolutional architectures (e.g., TextCNN) in trimodal settings, to the global dependency modeling of large-scale transformers (e.g., ViLBERT) in VL tasks. To further substantiate that the inverse effectiveness mechanism captures a fundamental learning dynamic independent of the underlying topology, we extend our evaluation to the SNN domain and verify IEMF across two distinct backbones: spiking ResNet and spiking transformers.

#### Convolutional architectures (ResNet-based)

**Fig. 5.**
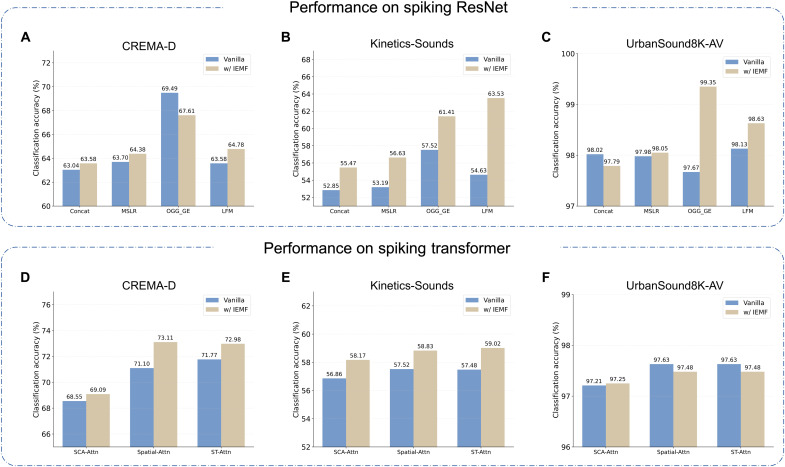
Architectural generalizability and performance improvement of the proposed IEMF across diverse SNN backbones. Performance on spiking ResNet. We evaluate the proposed method across four distinct fusion strategies (Concat, MSLR, OGM_GE, and LFM) on three benchmarks: CREMA-D (**A**), Kinetics-Sounds (**B**), and UrbanSound8K-AV (**C**). The IEMF-augmented models (khaki bars) outperform the vanilla baselines (blue bars), validating the efficacy of the inverse effectiveness mechanism in convolutional architectures. Performance on spiking transformer. To verify the universality of the approach, we extend the evaluation to attention-based backbones with three cross-attention mechanisms (SCA-Attn, Spatial-Attn, and ST-Attn) on CREMA-D (**D**), Kinetics-Sounds (**E**), and UrbanSound8K-AV (**F**). The accuracy gains demonstrate that the IEMF strategy is model-agnostic and robust to underlying network topologies (CNN versus transformer).

We first verify the robustness of IEMF within standard convolutional backbones. As shown in Fig. 5 (A to C), IEMF consistently improves performance across four distinct fusion strategies (Concat, MSLR, OGG_GE, and LFM). This robustness is particularly critical for neuromorphic computing, where traditional fusion methods often struggle with the sparsity of spiking streams. For instance, on the Kinetics-Sounds dataset using the LFM fusion method, the baseline SNN accuracy was 54.63%, lagging behind its ANN counterpart (55.28%). However, by introducing IEMF, the SNN accuracy surged to 63.53%, surpassing the ANN baseline. This result confirms that IEMF can effectively compensate for information loss in sparse, event-driven representations without requiring architectural changes.

#### Transformer-based architectures (cross attention-based)

To rigorously validate applicability across fundamentally different fusion paradigms, we extended our evaluation to spiking transformers equipped with cross-attention mechanisms. We specifically investigated three distinct variants: Spiking Cross Attention (SCA) (*41*), spatial, and spatial-temporal (*51*) cross-attention to verify IEMF’s robustness across varying granularities of feature interaction. This represents a significant shift from the direct feature aggregation typical of CNNs to the relational dependency modeling via attention maps in transformers. As detailed in Fig. 5 (D to F), IEMF yields gains in this distinct paradigm without requiring hyperparameter recalibration. Specifically, on the CREMA-D dataset, IEMF improved top-1 accuracy from 71.10 to 73.11% with spatial cross-attention, and from 71.77 to 72.98% with spatial-temporal cross-attention. These results, achieved using the exact same configuration as in CNNs, provide compelling evidence that the inverse effectiveness mechanism is invariant to the underlying integration topology and genuinely architecture agnostic.

### Comparison with gating-based fusion methods

To explicitly verify that the performance gains of IEMF arise from optimization-level feature refinement rather than simple inference-time heuristics, we benchmarked our method against two inference-time reliability gating baselines: (i) entropy-based Shannon gating, which selects modalities based on the lowest predictive entropy, and (ii) confidence-based mixture-of-experts (MoE) gating, which dynamically ensembles branches using softmax confidence scores.

#### Mechanistic distinction: Optimization versus selection

**Fig. 6.**
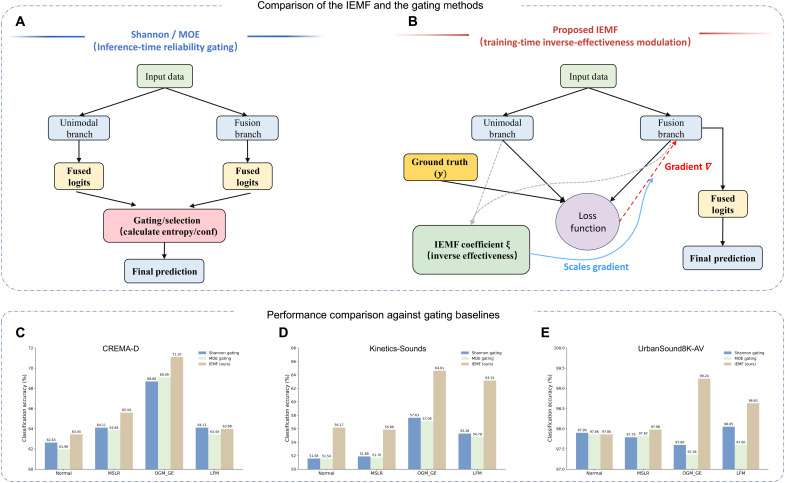
Conceptual distinction and empirical superiority of the proposed IEMF over traditional reliability gating baselines. Conceptual comparison: Inference-time reliability gating versus training-time IEMF. (**A**) Traditional strategies (e.g., Shannon/MoE) that operate at inference time by dynamically selecting or weighting outputs based on uncertainty metrics. (**B**) The proposed IEMF, which functions strictly at training time. It uses the inverse effectiveness coefficient ξ derived from ground truth to modulate the backward gradients ∇, reshaping the optimization trajectory without introducing any computational overhead during inference. Quantitative comparison: IEMF versus reliability-gating baselines. Classification accuracy across three benchmarks: (**C**) CREMA-D, (**D**) Kinetics-Sounds, and (**E**) UrbanSound8K-AV. The proposed IEMF (brown bars) consistently outperforms the inference-time gating baselines (Shannon and MoE, represented by blue and green bars, respectively). These results demonstrate that modulating learning dynamics yields superior representations compared to post hoc reliability gating.

As illustrated in Fig. 6 (A and B), these empirical results underscore a fundamental methodological divergence. Reliability gating methods operate as inference-time heuristics, functioning as post hoc filters that dynamically reweight outputs based on uncertainty. While this strategy mitigates noise impact, it leaves the underlying feature representations unchanged. In contrast, IEMF intervenes strictly at training time by modulating gradient backpropagation via the inverse effectiveness principle, thereby imposing zero additional computational overhead during inference. This mechanism fundamentally reshapes the optimization landscape, compelling the model to extract complementary features from weak modalities rather than merely suppressing them. The superior performance of IEMF validates that the observed gains stem from learning intrinsically robust joint representations, an optimization benefit that extends beyond the capability of superficial decision-level gating.

#### Quantitative superiority

As presented in Fig. 6 (C to E), IEMF consistently outperforms both gating strategies across all three datasets (CREMA-D, Kinetics-Sounds, and UrbanSound8K-AV). The performance gap is particularly pronounced in challenging scenarios where visual or audio cues are degraded. For instance, under the OGM_GE fusion strategy on the Kinetics-Sounds dataset, the MoE gating baseline yields an accuracy of 57.09%, comparable to the Shannon gating (57.63%). In stark contrast, IEMF boosts the accuracy to 64.61%, representing a relative improvement of more than 13%. Similarly, on CREMA-D with OGM_GE fusion method, IEMF achieves 71.10%, surpassing both Shannon gating 68.68% and MoE gating 69.09%. Notably, we observed that Shannon gating performance effectively collapses to that of the vanilla baseline. Analysis reveals that in the well-trained models, the predictive entropy of the multimodal branch is consistently lower than that of individual unimodal branches. Consequently, the Shannon gating mechanism trivially defaults to selecting the fused output for nearly all samples.

### Ablations and analysis

To further validate IEMF’s effectiveness and understand its internal mechanisms, we conducted comprehensive ablation studies and diagnostic analyses.

#### Mechanism ablation

**Fig. 7.**
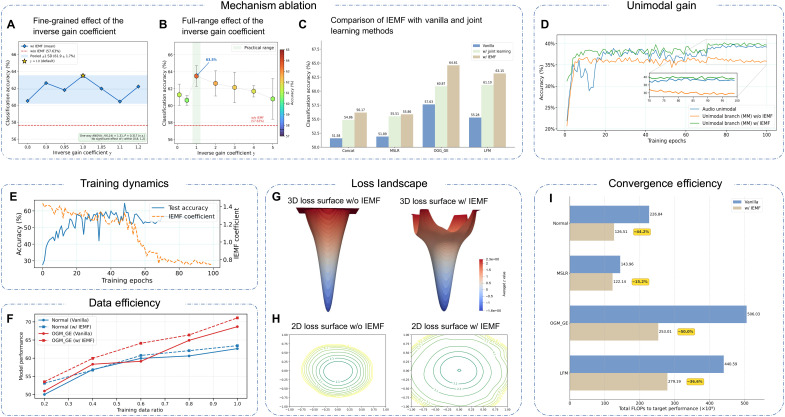
Comprehensive mechanism analysis, ablation studies, and efficiency benefits of IEMF. Mechanism ablation. (**A**) Fine-grained sensitivity of γ within [0.8, 1.2]: All seven configurations fall within the pooled ±1 SD band, and a one-way ANOVA confirms no statistically significant effect. (**B**) Effect of γ over the full range: Performance peaks at γ=1, remains above the baseline without IEMF (dashed red line), and declines monotonically at extreme values. (**C**) Removing the IEMF term (“Joint Learning only”) leads to a clear performance drop across all fusion strategies, highlighting the essential role of IEMF. (**D**) Unimodal gain analysis. Test accuracy for a unimodal model trained alone, the audio branch from a multimodal model without IEMF, and with IEMF. IEMF yields a persistent relative gain for the unimodal branch. (**E**) Dynamics of the IEMF coefficient. Evolution of ξ (dashed orange) alongside test accuracy (solid blue) during training. ξ starts high to accelerate multimodal integration and decreases as the network converges, maintaining fusion stability. (**F**) Data efficiency analysis. Performance comparison of the ANN model on CREMA-D under normal and OGM_GE settings across varying training data ratios (20 to 100%). Loss landscape visualization. (**G**) 3D loss surface comparison: vanilla method (left) versus IEMF-enhanced method (right). (**H**) 2D contour plots: without IEMF versus with IEMF (e3). The IEMF method leads to broader and flatter minima. (**I**) Convergence efficiency analysis. Comparison of total FLOPs required to reach target performance between standard models (blue) and IEMF-enhanced models (brown). IEMF substantially reduces computational costs across all fusion methods, with reductions ranging from 15.2 to 50.0% (highlighted in yellow percentages). n.s., not significant.

To further validate IEMF’s effectiveness, we conducted mechanism ablation experiments (Fig. 7, A and B). In Fig. 7B, we analyzed classification accuracy changes under different inverse gain coefficient γ settings. Results show appropriate inverse gain effectively improves model performance, with optimal accuracy at γ=1, indicating IEMF effectively balances unimodal and multimodal fusion signal contributions to maximize dynamic compensation. With larger coefficients (e.g., γ=5), accuracy decreases, likely because of training instability from excessive modulation intensity. Conversely, without IEMF (w/o IEMF baseline in Fig. 7B), classification accuracy is notably lower than all inverse gain coefficient settings, further validating the crucial role of IEMF in enhancing multimodal fusion. In Fig. 7C, we compared baseline models (vanilla), models with joint learning strategy, and IEMF-enhanced models. We specifically included joint learning comparison to systematically evaluate IEMF’s effectiveness. Joint learning adds independent classification heads for each modality without introducing new modalities, enhancing unimodal feature discriminability. IEMF dynamically modulates fusion module updates based on unimodal-fusion modality strength relationships for more refined compensation, mechanistically different approaches. Results show that while joint learning provides performance improvements, IEMF further enhances model performance, validating IEMF’s superior generalizability through dynamic fusion module adjustment in existing multimodal learning frameworks.

#### *Fine-grained* γ *sensitivity analysis*

To more thoroughly characterize the sensitivity of IEMF to the inverse gain coefficient γ, we conducted additional experiments on CREMA-D at thirteen γ values, including fine-grained steps {0.8,0.9,0.95,1.0,1.05,1.1,1.2}, each repeated across three independent random seeds. As shown in Fig. 7A, every γ value within [0.8, 1.2] falls within the pooled ±1 SD confidence band, and a one-way analysis of variance (ANOVA) confirms no statistically significant effect of γ [F(6,14)=1.31, P=0.317]. Over the full range [0.1, 5.0] (Fig. 7B), performance peaks at the default γ=1.0 and declines monotonically at extreme values, consistent with the mathematical bound $\xi_{t}\in \left(0,2\gamma \right)$. These results confirm that performance near γ=1.0 is continuous (no cliff-like drops), the practical range [0.8, 1.2] constitutes a robust plateau well above the baseline, and mild task-specific tuning of γ within this range is possible but not necessary as the default γ=1.0 already provides robust gains across all evaluated benchmarks.

#### Unimodal gain analysis

We further evaluated how multimodal learning affects performance of weaker modality branches (audio) on Kinetics-Sounds using OGM_GE fusion (Fig. 7D). After multimodal training, we fine-tuned the audio branch to analyze fusion effects on unimodal perception. Results show that traditional fusion methods (orange curve) lead to overfitting, limiting performance gains and even underperforming independently trained unimodal branches (blue curve). This suggests that modal interference in conventional fusion degrades unimodal feature quality and perception. In contrast, with IEMF (green curve), the unimodal branch maintains higher, more stable accuracy throughout training with significant early performance advantages. This confirms that IEMF not only optimizes multimodal fusion but effectively mitigates modal interference, promoting better unimodal feature learning and generalization.

#### Training dynamics

Figure 7E shows the test-set evolution of IEMF dynamic coefficients ξ and classification accuracy across training epochs. IEMF adapts fusion module behavior based on fusion effectiveness: During early training, fusion benefits are greater, keeping dynamic coefficient ξ high to maximize multimodal advantages; as unimodal features mature and fusion advantages diminish, ξ naturally decreases, reflecting reduced fusion dependency and helping maintain module stability for consistent test performance.

#### Self-correcting dynamics of the IEMF coefficient

An important property of IEMF is that the coefficient $\xi_{t}$ exhibits a self-correcting negative feedback behavior that prevents the fusion module from stalling. When unimodal branches mature rapidly ($S_{t}^{a0v}\gg S_{t}^{\mathrm{av}}$), $\xi_{t}$ decreases and the fusion module receives attenuated gradients, temporarily slowing its learning while the unimodal encoders continue to update normally. As unimodal representations improve, the fusion module receives higher-quality inputs, which in turn increases $S_{t}^{\mathrm{av}}$ and pulls $\xi_{t}$ back upward, forming a self-stabilizing loop. Moreover, because the fusion branch takes both unimodal features as input, in practice, $S_{t}^{\mathrm{av}}\geq S_{t}^{a0v}$ once training has progressed sufficiently, yielding a practical lower bound $\xi_{t}\geq \gamma \cdot \left[1+\text{tanh}\left(0\right)\right]=\gamma >0$. This analysis is consistent with the empirical trajectory of $\xi_{t}$ shown in Fig. 7E, where the coefficient stabilizes at a moderate positive value and never approaches zero, while both unimodal and fusion branches benefit simultaneously (Fig. 7B), confirming the absence of any learning freeze effect. This self-regulating property ensures that IEMF maintains a balanced optimization dynamic throughout training without requiring manual scheduling.

#### Data efficiency

To investigate the robustness of IEMF under data-constrained scenarios, we evaluated model performance on the CREMA-D dataset across varying training data ratios (20 to 100%), as shown in Fig. 7F. The results demonstrate that IEMF consistently outperforms the vanilla baseline across all data scales. Notably, the performance gap is most pronounced in low-resource regimes (e.g., at the 20% ratio), where the vanilla model suffers significant degradation while the IEMF-enhanced model maintains robust accuracy. This indicates that our method effectively mitigates the dependency on large-scale annotated data, leveraging the inverse effectiveness mechanism to maximize feature utilization even when training samples are scarce.

#### Loss landscape analysis

To validate the generalization properties of our proposed method, we visualized the loss landscapes of models with and without IEMF as shown in Fig. 7 (G and H). The three-dimensional (3D) loss surface visualization illustrated in Fig. 7G reveals significant topological differences: The baseline method exhibits a sharper, cone-like minimum, while the IEMF-enhanced model displays a broader, more gradual basin structure. This distinction is further emphasized in the 2D contour plots depicted in Fig. 7H: Without IEMF, the contours form elongated elliptical patterns, indicating inconsistent curvature across different parameter directions; with IEMF, contours appear more circular and uniformly spaced, confirming a markedly flatter minimum region. These observations closely align with our subsequent theoretical analysis presented later here, which demonstrates that IEMF directs the optimization process toward flatter regions of the loss landscape, a characteristic associated with the improved generalization performance observed in our experimental results.

#### Complexity and efficiency analysis

To provide a holistic assessment of algorithmic efficiency, we analyzed IEMF from two distinct perspectives: hardware-agnostic resource consumption and learning efficiency. First, regarding resource consumption, we decoupled computational cost from performance as detailed in table S10 (see the Supplementary Materials for full data). Compared to the vanilla baseline, IEMF introduces negligible overhead. Specifically, the increases in model parameters (+0.03 M) and GPU memory usage (+12 MB) are less than 0.2%, and the floating-point operations (FLOPs) remain virtually unchanged because of the lightweight nature of the inverse effectiveness module. While the total training time sees a slight increase (from 2.85 to 2.96 hours), the inference time remains identical to the baseline (9.74 s), ensuring no latency penalty during deployment. Concretely, the additional computation introduced by IEMF comes exclusively from computing the batch-level modality-strength scores $S_{t}^{a-v}$ and $S_{t}^{\mathrm{av}}$, which involves extracting ground truth–indexed confidence scores and performing a batch-level averaging operation with complexity O(B⋅C) (where B is the batch size and C is the number of classes), comparable in magnitude to a single batch normalization layer. This corresponds to a +3.9% per-epoch wall-clock overhead on a single NVIDIA A100 GPU, and critically, IEMF operates exclusively during training; the coefficient computation is disabled at test time, ensuring identical inference latency to the vanilla baseline (9.74 s; table S10).

Second, beyond these statistics, we evaluated the total training budget required to reach optimal performance, termed as convergence efficiency in Fig. 7I. Since IEMF maintains a reasonable per-epoch complexity (as verified above), its ability to accelerate convergence directly translates to computational savings. Across all fusion methods, IEMF consistently reduces the total training budget (calculated as epochs to target × FLOPs) by significant margins, ranging from 15.2% for MSLR to 50.0% for OGM_GE. Normal and LFM configurations also show reductions of 44.2 and 36.6%, respectively. By dynamically modulating fusion behavior, IEMF achieves a superior trade-off: It combines the low per-step overhead of lightweight modules with the high learning efficiency of faster convergence, optimizing resource utilization for real-world applications.

In summary, IEMF demonstrates consistent performance improvements across datasets and fusion strategies. Systematic experiments validate its effectiveness in dynamically regulating fusion, mitigating modal interference, enhancing unimodal learning, and improving robustness, providing an efficient and well-generalizing fusion strategy for multimodal perception tasks.

## DISCUSSION

### Biological insights into multimodal integration

Despite significant advances in multimodal fusion, many key biological principles have not been fully explored and applied in artificial intelligence systems, which could further enhance the robustness and adaptability of multimodal systems. In this study, we propose a brain-inspired deep neural network multimodal integration method based on the inverse effectiveness mechanism observed in biological systems, providing verifiable theoretical foundations and methodological support for multimodal information integration. Specifically, we propose an IEMF method that dynamically adjusts the weights of modal fusion modules based on the relationship between the strength of single-modal cues and the modality strength after modal fusion. Our approach systematically considers the complementary interactions between modalities, considerably improving not only the performance and generalization capabilities of multimodal systems but also their computational efficiency. This dual advantage, enhanced robustness coupled with reduced computational costs, offers insights into why inverse effectiveness might have evolved as a critical mechanism in biological systems, where both perceptual reliability and metabolic efficiency are under evolutionary pressure.

In the IEMF framework, we prioritize inverse effectiveness in the network training process by introducing an inverse effectiveness gain coefficient that applies gradient regulation to fusion weights, naturally forming an internal bias of “weak modality-high gain, strong modality-low gain” during the learning phase. This strategy aligns with the physiological mechanism of cross-modal experience shaping plasticity in early neural development in biological organisms, where newborn individuals initially lack multisensory integration abilities. These abilities are not innate but develop through continuous shaping of neural circuits via early cross-modal experiences, adapting to the environment and optimizing multimodal integration performance (*52*). Furthermore, we apply inverse effectiveness–driven fusion strategies uniformly across all input channels of the model, consistent with the view in Regenbogen *et al.* (*34*) that inverse effectiveness manifests not only under degraded stimulus conditions but also with clear stimuli.

This research confirms the long-standing intuition that how modalities are combined is as important as how many are combined. By introducing inverse effectiveness rules from cortical circuits into gradient-based optimization learning systems, we achieved (i) effective generalization in both ANN and SNN models; (ii) significant performance improvements in audiovisual classification, audiovisual continual learning, and AVQA tasks; and (iii) substantial computational efficiency gains with up to 50% reduction in computational costs across diverse fusion methods. Systematic experiments demonstrate that after integrating the IEMF mechanism into existing multimodal methods, models achieved performance superior to original state-of-the-art techniques across various multimodal tasks, further indicating that introducing bioinspired mechanisms can effectively improve the efficiency of multimodal integration, expanding its potential in artificial intelligence applications. These findings not only highlight the advantages of incorporating biological principles into machine learning models but also provide new directions for future research in neuromorphic computing and multisensory integration.

### Other biological mechanisms of multimodal integration

It is worth discussing that while this research emphasizes the importance of inverse effectiveness in multimodal integration, there are two other equally important principles in multimodal biological perception processes: temporal congruence and spatial congruence. These mechanisms are particularly important in dynamic multimodal integration. Temporal congruence refers to visual and auditory inputs maintaining coordination in time, thereby optimizing perceptual and decision-making performance. Experimental studies (*53*, *54*) demonstrate that when visual and auditory stimuli are presented in close synchronization within a 0- to 200-ms time window, they enhance the accuracy and reaction speed of perceptual judgments. In contrast, temporal asynchrony leads to decreased activation intensity in relevant brain regions, weakening the integration effect. Spatial congruence refers to different sensory modalities maintaining consistency or proximity in spatial location, thereby enhancing the joint representation of cross-modal signals. Research has found that multisensory neurons (such as neurons in the superior colliculus) exhibit integration enhancement effects only when audiovisual stimuli originate from the same or adjacent spatial locations; otherwise, integration may be inhibited or show no integration response (*55*). For example, in real-world scene understanding, object recognition, and tracking tasks, accurately matching sound sources with corresponding visual objects is key to successful multimodal perception. Temporal and spatial congruence are crucial for accurate multimodal integration.

Although temporal and spatial congruence are indispensable in biological perception, given that the tasks selected in this study inherently have strong input synchronization characteristics (i.e., dual-modal inputs from the same source at the same moment), we did not explicitly model these mechanisms in the current work. Specifically, the sensory inputs in this study’s tasks naturally have synchronization and correspondence relationships; therefore, these congruence factors have already been implicitly considered in the multimodal fusion process. Looking forward, further research could explore how to explicitly incorporate temporal and spatial congruence into the IEMF framework by introducing asynchronous, spatially disparate multimodal input samples, thereby training models to effectively integrate under more complex temporal and spatial variation conditions, further advancing biologically inspired multimodal learning systems toward broader application domains.

### Distinctions from adaptive balancing and uncertainty-based approaches

Our work aligns with the emerging consensus in multimodal learning that static fusion strategies are insufficient for complex, dynamic environments. Recent adaptive approaches, such as OGM-GE (*4*) and DynCIM (*23*), have successfully demonstrated that dynamically modulating optimization trajectories can mitigate modality imbalance. We share the critical insight driving these works: The relative importance of modalities and the intensity of their interaction should not be maintained as constants throughout the training process.

However, IEMF diverges fundamentally from these methods in its underlying mechanism and objective. Traditional uncertainty-based or confidence-aware gating methods (*4*, *21*, *22*) typically follow a “reliability-gating” logic: They down-weight or suppress modalities with high uncertainty to protect the decision boundary. While effective for filtering noise, this strategy can lead to information loss when all modalities are imperfect. In contrast, inspired by the biological principle of inverse effectiveness, IEMF adopts a “compensatory” logic. Instead of suppressing the learning of weak modalities, IEMF actively accelerates the gradient updates of the fusion module when unimodal signals are weak. This forces the network to find deeper, nontrivial interactions between modalities to compensate for the lack of strong unimodal features rather than merely selecting the “safest” input.

Crucially, IEMF is designed not merely as a competitor to existing architectures but as a generalized optimization mechanism that can be seamlessly integrated into them. First, as demonstrated in our experimental results (e.g., Fig. 2, A to C), IEMF was successfully applied on top of MSLR, OGM-GE, and LFM, consistently boosting their performance. This confirms that optimizing the fusion gradient flow (IEMF) provides benefits orthogonal and complementary to balancing unimodal encoders (OGM-GE). Second, to explicitly validate the superiority of our strategy over static reliability judgments, we compared IEMF against standard gating baselines (Shannon information gating and confidence-based MoE) in the Fig. 6 and table S8. The empirical data show that IEMF’s dynamic, training-time gradient modulation yields more robust joint representations than simply gating modalities based on confidence scores. Last, unlike dynamic gating mechanisms that rely on explicit reliability estimation and reweighting during inference, IEMF allows the model to internalize robust fusion strategies directly into the weights during training, requiring no additional computation steps at test time.

### Limitations and boundary analysis

#### Optimization trade-offs in high-congruence regimes

While IEMF demonstrates superior robustness in complex environments, we acknowledge that in scenarios characterized by extremely high modality congruence and low noise, the method may exhibit marginal performance degradation compared to vanilla baselines. We attribute this to an optimization-regularization trade-off: Simple baselines benefit from direct gradient paths in these idealized settings, whereas the adaptive mechanisms in IEMF introduce minor optimization complexity. Consequently, the model maintains statistical parity with the baseline in such ideal conditions, implying that any observed marginal differences fall within the range of normal experimental variance. Notably, our extended analysis (see table S11) demonstrates that despite these minor individual variances, the method yields statistically significant improvements on average, and any residual drops can be mitigated by adjusting the regularization sensitivity (γ), ensuring that stability in noisy deployments does not come at the expense of performance in clean regimes.

#### Challenges in extreme real-world settings

Furthermore, although IEMF is designed to mitigate noise, its efficacy is bound by the quality of the available signals in practical applications. First, regarding missing data: Our current implementation assumes the presence of all modality inputs to calculate reliability scores. In real-world sensors where entire streams may drop out (e.g., camera occlusion or microphone failure), the inverse effectiveness calculation would require modification (e.g., imputation or dynamic masking) to prevent mathematical errors, a scope not covered in this work. Second, regarding catastrophic noise: The core premise of inverse effectiveness is that weak cues in one modality can be compensated by others. However, in “blackout” scenarios where all modalities are simultaneously corrupted by severe noise (approaching zero signal-to-noise ratio), the mechanism cannot recover meaningful information as there is no “effective” anchor to drive the fusion. Future work will focus on extending IEMF to handle asynchronous missing modalities and incorporating self-supervised priors to survive total-modality degradation.

### Broader applications in scientific multimodal settings

Beyond the benchmark tasks evaluated in this work, IEMF is well suited to scientific multimodal settings where participating modalities differ substantially in signal-to-noise ratio, data abundance, or annotation quality. We highlight four representative biological scenarios where IEMF’s compensatory logic can be effectively applied.

#### Single-cell multi-omics integration 

Joint profiling of transcriptomics [single-cell RNA sequencing (scRNA-seq)] and chromatin accessibility (scATAC-seq) is a rapidly growing frontier in single-cell genomics (*56*). The two modalities exhibit a pronounced asymmetry in per-feature informativeness: scATAC-seq data are extremely sparse because of cell type–specific chromatin states and the inherent diploid limit, while scRNA-seq shows substantially higher per-feature detection rates (*57*). Recent benchmarks (*58*) confirm that integration methods often perform notably worse on the sparser ATAC component, and recent deep-learning approaches (*59*, *60*) have begun to adopt end-to-end joint optimization across both modalities. IEMF would naturally amplify fusion learning when the ATAC modality provides weak discriminative evidence while tempering it when transcriptomic signals alone are already informative.

#### Cancer survival prediction

In computational oncology, whole-slide images provide rich morphological detail but, under the multiple instance learning paradigm, the slide-level supervision signal is diluted across thousands of patches, weakening the effective per-sample discriminative contribution of histological features during training relative to genomic profiles. Recent work has explicitly modeled this as an asymmetric fusion problem (*61*), and complementary studies have demonstrated the value of integrating histology with genomics even under partial modality availability (*62*, *63*). IEMF’s compensatory logic would actively strengthen fusion when histological evidence is ambiguous, promoting the discovery of nontrivial pathology—genomics correspondences.

#### Neuroimaging fusion

Electroencephalography and functional magnetic resonance imaging offer complementary spatiotemporal views of brain dynamics (*64*, *65*), but the signal quality of each varies substantially across sessions and subjects because of distinct artifact profiles. IEMF’s batch-adaptive modulation coefficient $\xi_{t}$ would respond to this sample-level variation, automatically increasing the fusion learning rate for mini-batches where one modality is degraded while maintaining moderate updates when both channels are reliable. Its zero inference-time overhead is particularly attractive for latency-sensitive applications such as real-time neurofeedback.

#### Protein function prediction

Protein sequence data are abundant and well-served by pretrained protein language models such as ESM-2 (*66*), while experimentally determined structures remain far scarcer. Computationally predicted structures (e.g., via AlphaFold) vary widely in confidence, particularly in flexible loop regions and intrinsically disordered regions. This heterogeneous reliability can create a modality imbalance where sequence features dominate joint training. Leveraging structure-aware representations alongside sequences has been shown to improve function prediction (*67*). IEMF would amplify fusion learning for samples where structural information provides unique complementary evidence (e.g., binding pockets or allosteric sites), while its convergence acceleration would reduce the computational cost of training on large-scale structure–sequence pairs.

In each scenario, the central challenge aligns with IEMF’s design principle: Modalities carry asymmetric and dynamically varying information quality, and effective fusion requires proportionally greater integrative effort when individual channels are weak.

## MATERIALS AND METHODS

### Neuron models in ANNs and SNNs

In neural networks, the information flow is governed by the dynamics of neuronal activation. In this work, we adopt two distinct neuron models: the continuous artificial neurons used in ANNs and the spike-based neurons in SNNs.

In ANNs, information is processed continuously. Each neuron computes a weighted sum of its inputs and applies a nonlinear activation function to produce its output y=f(Wx+b), where x is the input vector, W is the weight matrix, b is the bias term, and f(⋅) denotes a nonlinear function such as ReLU (*68*) or sigmoid.

In contrast, SNNs more closely mimic biological neurons by communicating via discrete spike events. We use the widely used leaky integrate-and-fire (LIF) model (*69*) to capture the membrane potential dynamics. Upon receiving a synaptic input current I(t), the membrane potential U(t) accumulates over time. When U(t) crosses a threshold $U_{\text{th}}$, a spike is emitted and the potential is reset to the resting value $U_{\text{rest}}$. The continuous-time dynamics of the LIF neuron are given by

$$\tau_{m}\frac{\mathrm{dU}\left(t\right)}{\mathrm{dt}}=-\left[U\left(t\right)-U_{L}\right]-\frac{g_{E|I}}{g_{L}}\left[U\left(t\right)-U_{E|I}\right]+\frac{I_{s}}{g_{L}}$$ (1)

where $\tau_{m}=C_{m}/g_{L}$ is the membrane time constant, $C_{m}$ is membrane capacitance, $g_{L}$ is the leak conductance, and $g_{E|I}$ and $U_{E|I}$ denote the conductance and reversal potentials for excitatory or inhibitory synapses. $I_{s}$ is the synaptic input current. To simplify the formulation, we aggregate the synaptic terms into an effective input current $\mathrm{RI}\left(t\right)≜-\frac{g_{E|I}}{g_{L}}\left[U\left(t\right)-U_{E|I}\right]+\frac{I_{s}}{g_{L}}$, reducing the membrane potential equation to

$$\tau_{m}\frac{\mathrm{dU}\left(t\right)}{\mathrm{dt}}=-\left[U\left(t\right)-U_{\text{rest}}\right]+\mathrm{RI}\left(t\right)$$ (2)

For numerical simulation, we set $U_{\text{rest}}=0$ and discretize the above dynamics. To clearly distinguish continuous and discrete states, we denote membrane potential as $u^{t}$ at discrete time step t. The complete discrete-time update of the membrane potential and spike generation at layer l is

$$\begin{array}{c} u_{\text{pre}}^{t,l}=\tau u^{t-1,l}+W^{l}s^{t,l-1}\;\left(\text{accumulation}\right)\\ s^{t,l}=H\left(u_{\text{pre}}^{t,l}-u_{\text{th}}\right)\;\left(\text{spike firing}\right)\\ u^{t,l}=u_{\text{pre}}^{t,l}\left(1-s^{t,l}\right)\;\left(\text{reset mechanism}\right) \end{array}$$ (3)

where $W^{l}$ is the weight matrix from layer l−1 to l, $s^{t,l-1}$ denotes the spike train from the previous layer at time t, $\tau =1-\frac{1}{\tau_{m}}$ is the leak factor controlling the temporal decay of the membrane potential, and H(⋅) is the Heaviside step function used to generate binary spike outputs.

### Multimodal integration formulation

We denote a multimodal input as $x=\left(x^{a},x^{v}\right)$, where $x^{a}\in X^{a}$ and $x^{v}\in X^{v}$ represent inputs from two modalities (e.g., audio and visual). These are independently processed by two encoders $\varphi^{a}\left(\cdot ;\theta^{a}\right)$ and $\varphi^{v}\left(\cdot ;\theta^{v}\right)$ to obtain modality-specific latent representations

$$z^{a}=\varphi^{a}\left(x^{a};\theta^{a}\right),\;z^{v}=\varphi^{v}\left(x^{v};\theta^{v}\right)$$ (4)

where $\theta^{a}$ and $\theta^{v}$ represent the trainable parameters of the audio and visual encoders, respectively. The extracted features are fused using a general fusion operator F(⋅,⋅), i.e., $z^{\text{av}}=F\left(z^{a},z^{v}\right),$ followed by a classifier $h\left(\cdot ;\theta^{h}\right)$ that maps the fused audio visual features to a prediction $\hat{y}=h\left(z^{\text{av}};\theta^{h}\right),$ where $\theta^{h}$ denotes the classifier parameters.

In the widely adopted vanilla fusion strategy, such as feature concatenation, the fusion operator takes the form

$$F\left(z^{a},z^{v}\right)=W^{f}\left[z^{a};z^{v}\right]+b^{f}$$ (5)

where [⋅;⋅] denotes the concatenation operation, $W^{f}\in {\mathbb{R}}^{M\times \left(d_{a}+d_{v}\right)}$ and $b^{f}\in {\mathbb{R}}^{M}$ are the parameters of the fusion layer, and M is the number of output classes.

The multimodal learning goal is to train a multimodal model $f_{\theta }:X^{a}\times X^{v}\to Y$, where the learnable parameters $\theta =\left\{\theta^{a},\theta^{v},\theta^{h}\right\}$, that minimizes the empirical risk over a dataset $D={\left(x_{i}^{a},x_{i}^{v},y_{i}\right)}_{i=1}^{N}$. The training objective is

$$\text{arg}{\text{min}}_{\theta }\;L\left(\theta \right)=\frac{1}{N}\sum_{i=1}^{N}L_{\text{ce}}\left[f_{\theta }\left(x_{i}^{a},x_{i}^{v}\right),y_{i}\right]$$ (6)

where $L_{\text{ce}}\left(\cdot \right)$ denotes the cross-entropy loss function.

### Inverse effectiveness–driven multimodal fusion

Most previous studies have focused on designing sophisticated fusion blocks, refining the joint representation $z^{\text{av}}$ to enhance cross-modal interactions, yet they rarely consider how the relative informativeness of each unimodal stream relative to the fused output should guide the fusion module. In this work, we draw inspiration from the principle of inverse effectiveness. Instead of using static cross-modal integration, we adaptively adjust the update rate of the fusion module’s weights by contrasting the estimated information content of each unimodal branch with that of the integrated multimodal representation.

First, for each sample i in a mini-batch $B_{t}$, we evaluate the per-sample modal information content $c_{i}$ according to the following equation

$$\begin{array}{c} c_{i}^{a}={\left[p_{i}^{a}\right]}_{y_{i}},p_{i}^{a}=\pi \left(W_{t}^{a}\cdot z^{a}+b_{t}^{a}\right)\\ c_{i}^{v}={\left[p_{i}^{v}\right]}_{y_{i}},p_{i}^{v}=\pi \left(W_{t}^{v}\cdot z^{v}+b_{t}^{v}\right) \end{array}$$ (7)

where $W_{t}^{a/v}$, $b_{t}^{a/v}$ are the parameters of the audio/visual modal classification heads, respectively, and ${\left[p\right]}_{y_{i}}$ picks the probability assigned to the ground-truth label $y_{i}$. π is a normalization function, and here we choose the softmax function. The informativeness of the multimodal output is estimated in the same way

$$c_{i}^{\text{av}}={\left[p_{i}^{\text{av}}\right]}_{y_{i}},\;p_{i}^{\text{av}}=\pi \left(W_{t}^{\mathrm{av}}\cdot z^{\text{av}}+b_{t}^{\mathrm{av}}\right)$$ (8)

Next, we average the evaluated values in (*7*) and (*8*) to obtain the batch-level modality-strength scores

$$S_{t}^{a-v}=\frac{1}{2|B_{t}|}\sum_{i\in B_{t}}\left(c_{i}^{a}+c_{i}^{v}\right),\;S_{t}^{\mathrm{av}}=\frac{1}{|B_{t}|}\sum_{i\in B_{t}}c_{i}^{\mathrm{av}}$$ (9)

Following the biological observation that fusion should dominate and provides the greatest benefit when unimodal evidence is weak, we define an IEMF coefficient $\xi_{t}$. The IEMF coefficient quantifies how effective the unimodal branches are relative to the fused output. We map $\xi_{t}$ to a bounded value

$$\xi_{t}=\gamma \cdot \left[1+\kappa \;\left(1-\frac{S_{t}^{a-v}}{S_{t}^{av}}\right)\right],\;\gamma >0$$ (10)

where γ is the inverse gain coefficient controlling the overall magnitude of fusion modulation, and κ(⋅) denotes a generic bounded gating function; in this work, we instantiate κ with the hyperbolic tangent, i.e., κ(⋅)=tanh(⋅), owing to its smooth and symmetric saturation properties. Because κ(⋅)∈(−1,1), Eq. 10 confines the fusion coefficient to the interval $\xi_{t}\in \left(0,2\gamma \right)$.

IEMF coefficient $\xi_{t}$ magnitude varies intuitively with the strength ratio between unimodal and multimodal evidence:

1. Weak unimodal evidence $\left(S_{t}^{a-v}\ll S_{t}^{\mathrm{av}}\right)$. The term $\left(1-S_{t}^{a-v}/S_{t}^{\mathrm{av}}\right)$ is positive and thus $\left(\xi_{t}\right)$ approaches its upper bound. A larger $\xi_{t}$ amplifies the fusion gradient, encouraging the model to rely more heavily on cross modal integration.

2. Strong unimodal evidence $\left(S_{t}^{a-v}≳S_{t}^{\mathrm{av}}\right)$. As the ratio nears or exceeds 1, the inner term becomes nonpositive; 1+κ(⋅) decreases, pulling $\xi_{t}$ toward its lower limit 0. When the unimodal score predominates, a smaller $\xi_{t}$ attenuates the fusion update, preserving the integrity of an already robust unimodal pathway.

In summary, a large $\left(\xi_{t}\right)$ corresponds to weak unimodal cues and triggers a stronger adjustment of the fusion weights, whereas a small $\left(\xi_{t}\right)$ indicates confident unimodal predictions and results in a milder fusion update.

The IEMF coefficient $\left(\xi_{t}\right)$ is applied only to the fusion parameters, and the unimodal branches are not affected. Concretely

$$W_{t+1}^{f}=W_{t}^{f}-\eta \;\xi_{t}\;\nabla_{W^{f}}L\left(W_{t}^{f}\right)$$ (11)

where η is the learning rate and $\nabla_{W^{f}}L\left(W_{t}^{f}\right)$ is the raw fusion gradient. Because $0<\xi_{t}<2\gamma$, this scaling never reverses the descent direction, thereby maintaining optimization stability. Together, Eqs. 7 to 11, inspired by the inverse effectiveness principle, are characterized as: The fusion pathway receives a larger update when unimodal evidence is weak and a smaller one when unimodal confidence is high. This self-balancing rule enhances robustness under noise and improves the model’s generalization across diverse input conditions. The algorithmic steps of IEMF are summarized in algorithm S1.

### Numerical stability

The IEMF coefficient is inherently robust against numerical failure. First, each $c_{i}^{\mathrm{av}}$ is a softmax probability, which is strictly positive for all finite inputs, guaranteeing $S_{t}^{\mathrm{av}}>0$ unconditionally. Second, even if $S_{t}^{\mathrm{av}}\to 0^{+}$ hypothetically, the tanh gating saturates to −1, driving $\xi_{t}\to 0$ (attenuation) rather than +∞ (explosion); conversely, the upper bound $\xi_{t}<2\gamma$ is always finite. Third, to guard against floating-point underflow in FP16 mixed-precision training, we introduce an explicit tolerance $\epsilon =10^{-8}$ in the denominator of the IEMF coefficient (Eq. 10 and algorithm S1), consistent with standard practice in adaptive optimizers such as Adam (*70*). Together, these three properties, softmax positivity, tanh boundedness, and the ϵ safeguard, ensure that algorithm S1 is robust against gradient explosion under realistic training conditions.

### Practical guidelines for IEMF deployment

Drawing on our experimental results, we propose three practical heuristics for practitioners considering IEMF.

#### Heuristic~1 (modality imbalance diagnostic)

Before multimodal training, unimodal baselines are evaluated independently. If a substantial accuracy gap exists between the strongest and weakest modality, IEMF is strongly recommended as its adaptive gradient modulation amplifies fusion learning precisely when the weaker modality contributes limited discriminative evidence.

#### Heuristic~2 (noisy or degraded conditions)

When any input modality is subject to systematic noise, limited data, or quality degradation, IEMF’s compensatory mechanism provides significant robustness advantages. Our data-efficiency analysis (Fig. 7F and table S9) confirms that the performance gap between IEMF and the vanilla baseline widens as the training data ratio decreases.

#### Heuristic~3 (default-on recommendation)

In high-congruence, low-noise settings, IEMF maintains comparable or superior performance across all configurations (table S11). Given its negligible computational overhead (+3.9% training time, zero inference cost) and the mathematical guarantee that $\xi_{t}\to \gamma$ when modalities are balanced ($S_{t}^{a0v}\approx S_{t}^{\mathrm{av}}$), IEMF can be applied as a default training strategy with minimal risk.

In summary, if significant modality imbalance or noisy/degraded inputs exist, IEMF is strongly recommended; if modalities are balanced and clean, IEMF is safe to apply with negligible risk.

### Theoretical rationale for the IEMF optimization dynamics

In this section, we provide a theoretical rationale for the proposed IEMF. Our goal is to formally demonstrate how the IEMF coefficient, $\xi_{t}$, modulates the learning dynamics. This analysis serves to justify how the biologically inspired “inverse effectiveness” principle translates into a stable and adaptive update rule for deep neural networks. We prove that the inverse-effectiveness coefficient $\xi_{t}$ used by IEMF reduces the expected step size more in high-curvature directions, ensuring reliable convergence to local minima while maintaining optimization stability throughout the training process.

Assumption 1. The loss $L\left(W^{f}\right)$ is twice continuously differentiable, and there exist constants β,ρ>0 such that for all u,v, ‖∇L(u)−∇L(v)‖≤β‖u−v‖, ‖H(u)−H(v)‖≤ρ‖u−v‖. This means that the gradient and the Hessian are β-Smoothness and ρ-Lipschitz, respectively.

Theorem 1 (convergence properties of IEMF) Assume 1. Let $W^{f*}$ be a local minimizer and write $H^{*}=H\left(W^{f*}\right)$ with eigen-pairs ${\left[(\lambda_{i},e_{i})\right]}_{i=1}^{d}$, $0<\lambda_{1}\leq \ldots \leq \lambda_{d}$. The IEMF updates the fusion module parameters according to the Eq. 11. The deviation is defined $\left(\Delta_{t}≔W_{t}^{f}-W^{f*}=\sum_{i=1}^{d}\alpha_{i}^{t}e_{i}\right)$. A radius (*r* > 0) is chosen such that

$$\frac{\rho }{2}r<\lambda_{1}\;\text{and}\;\left\|\Delta_{t}\right\|\leq r\;\forall t$$ (12)

Then,

$$E\left[{\text{ηξ}}_{t}\lambda_{i}\right]\left\{ \begin{array}{cc} <{\text{ηλ}}_{i}, & S_{t}^{\mathrm{av}}/S_{t}^{\mathrm{av}}>x1\;\left(\text{unimodal dominated}\right),\\ ={\text{ηλ}}_{i}, & S_{t}^{\mathrm{av}}/S_{t}^{\mathrm{av}}=1,\\ >{\text{ηλ}}_{i}, & S_{t}^{\mathrm{av}}/S_{t}^{\mathrm{av}}<1\;\left(\text{fusion dominated}\right) \end{array}\right.$$ (13)

As a result, with IEMF, unimodal-dominated batches reduce sharp directions more than the vanilla method, whereas fusion-dominated batches allow at most a twofold increase in step size, thus preserving optimization convergence.

Proof: For any $W^{f}$ satisfying $\left\|W^{f}-W^{f*}\right\|\leq r$, we use ∇L(W), which denotes $\nabla_{W^{f}}L\left(W^{f}\right)$_,_ and construct $g\left(s\right)=\nabla_{W^{f}}L(W^{f*}+$_,_ +sΔ),s∈[0,1]. The fundamental theorem of calculus gives ∇L(W)= $g\left(1\right)-g\left(0\right)=\int_{0}^{1}\frac{d}{\mathrm{ds}}g\left(s\right)\mathrm{ds}$ . Then, we have

$$\nabla L\left(W\right)=\int_{0}^{1}H\left(W^{f}+s\Delta \right)\Delta \mathrm{ds}$$ (14)

Adding and subtracting $H^{*}\Delta$ inside the integral yields

$$\nabla L\left(W\right)=H^{*}\Delta +\int_{0}^{1}\left[H\left(W^{f*}+s\Delta \right)-H^{*}\right]\Delta \;\mathrm{ds}$$ (15)

We define the remainder term as

$$R\left(W\right)=\int_{0}^{1}\left[H\left(W^{f*}+s\Delta \right)-H^{*}\right]\Delta \mathrm{ds}$$ (16)

where the remainder R(W) can be estimated for an upper bound with the help of the ρ-Lipschitz condition

$$\left\|R\left(W\right)\right\|\leq \frac{\rho }{2}{\left\|W-W^{f*}\right\|}^{2}$$ (17)

which allows us to express the gradient as

$$\nabla L\left(W\right)=H^{*}\left(W-W^{f*}\right)+R\left(W\right)$$ (18)

If the above equation is inserted into the update (*11*) and the inner product is taken with $e_{i}$ and use ${\left(H^{*}e_{i}\right)}^{⊤}={\left(\lambda_{i}e_{i}\right)}^{⊤}$, we have

$$\alpha_{i}^{t+1}=\left(1-{\text{ηξ}}_{t}\lambda_{i}\right)\;\alpha_{i}^{t}-{\text{ηξ}}_{t}\;e_{i}^{⊤}R\left(W_{t}^{f}\right)$$ (19)

where $\alpha_{i}^{t}=e_{i}^{⊤}\Delta_{t}$. For the contraction argument, we need the last term ${\text{ηξ}}_{t}|e_{i}^{⊤}R\left(W_{t}^{f}\right)|$ to be strictly smaller than the linear part $\left(\lambda_{i}\left\|\Delta_{t}\right\|\right)$ even in the flattest direction $\left(\lambda_{i}=\lambda_{1}\right)$. With Cauchy-Schwarz formula and (*17*), we have

$$\begin{array}{cc} |e_{i}^{⊤}R\left(W_{t}^{f}\right)| & \leq \left\|R\left(W_{t}^{f}\right)\right\|,\\ \left\|R\left(W_{t}^{f}\right)\right\| & \leq \frac{\rho }{2}{\left\|\Delta_{t}\right\|}^{2} \end{array}$$ (20)

Applying the triangle inequality to Eq. 19 and substituting our derived bounds, we have

$$|\alpha_{i}^{t+1}|\;\leq \;|1-{\text{ηξ}}_{t}\lambda_{i}||\alpha_{i}^{t}|+{\text{ηξ}}_{t}\left(\frac{\rho }{2}{\left\|\Delta_{t}\right\|}^{2}\right)$$ (21)

By imposing $\frac{\rho }{2}r<\lambda_{1}$, we establish a crucial inequality

$${\text{ηξ}}_{t}\Delta_{t}\left(\frac{\rho }{2}{\left\|\Delta_{t}\right\|}^{2}\right)\leq {\text{ηξ}}_{t}\frac{\rho }{2}\;r\;\left\|\Delta_{t}\right\|<\xi_{t}\lambda_{1}\;\left\|\Delta_{t}\right\|$$ (22)

This inequality demonstrates that the quadratic remainder term is always strictly dominated by the linear term for all eigendirections i since $\lambda_{i}\geq \lambda_{1}$ for all i. Consequently, the convergence behavior of each component $\alpha_{i}^{t}$ is primarily determined by the multiplicative factor $\left(1-{\text{ηξ}}_{t}\lambda_{i}\right)$, with the remainder term providing a bounded perturbation that does not disrupt the overall convergence pattern established by the linear term. $\xi_{t}$ is computed by Eq. 10, with γ=1, $0<\xi_{t}<2$. Taking expectation of ${\text{ηξ}}_{t}\lambda_{i}$ over the mini-batch three cases arise, yielding exactly the inequalities stated in the theorem. □

Our analysis reveals two key aspects of IEMF’s convergence properties, primarily manifested through the factor $1-{\text{ηξ}}_{t}\lambda_{i}$, which precisely controls how quickly the error components $\alpha_{i}^{t}$ (representing the projection of parameter error onto each eigenvector) contract toward zero. In unimodal-dominated batches ($S_{t}^{anv}/S_{t}^{\mathrm{av}}>1$ resulting in $\xi_{t}<1$), the contraction factor satisfies $|1-{\text{ηξ}}_{t}\lambda_{i}|<|1-{\text{ηλ}}_{i}|$, meaning that high-curvature directions (larger $\lambda_{i}$ values) contract faster than in the vanilla method. Meanwhile, in fusion-dominated batches [$S_{t}^{anv}/S_{t}^{\mathrm{av}}<1$ resulting in $\xi_{t}\in \left(1,2\right)$], although step sizes may increase, they remain strictly bounded since $\xi_{t}<2$, ensuring the algorithm’s global stability. This dual mechanism, which balances a preference for minima with strict step-size constraints, ensures that IEMF maintains reliable convergence properties while adaptively adjusting step sizes.

Empirical studies have demonstrated a strong correlation between flatter minima and improved generalization performance (*71*, *72*). While Theorem 1 establishes the convergence of IEMF under standard smoothness assumptions, a stronger theoretical link between the geometric properties of the solution and its generalization remains challenging to formally prove. Nevertheless, we provide the following heuristic justification based on landscape sharpness analysis, supported by experimental observations.

Consider the sharpness of the loss landscape at a parameter configuration $W^{f}$, defined as

$$s\left(W^{f},\rho \right)≔{\text{max}}_{{|\epsilon |}_{2}\leq \rho }L\left(W^{f}+\epsilon \right)-L\left(W^{f}\right)$$ (23)

which quantifies the sensitivity of the loss to local perturbations. While we do not provide a formal guarantee, our empirical evidence and directional analysis suggest the following heuristic conclusion

Heuristic 1 (IEMF reduces landscape sharpness). Under dynamic training, IEMF adaptively reduces the step size in high-curvature directions. As a result, the sharpness

$$E\left[s\left(W^{f},\rho \right)\right]≲\alpha \cdot s_{\text{vm}}\left(W^{f},\rho \right)$$ (24)

where $s_{\text{vm}}$ is the sharpness observed under the vanilla method and α<1 is a factor that quantifies how much IEMF reduces the loss landscape’s sharpness through its adaptive modulation of optimization steps. This suggests that IEMF biases the optimization trajectory toward flatter regions of the loss landscape, a property that empirically correlates with improved generalization.

### Experimental settings and training details

#### Datasets

We divided the dataset as specified in the original dataset. Audiovisual classification: CREMA-D (*39*), an audiovisual dataset containing six most common emotion categories for speech emotion recognition with total 7442 video clips. We randomly divided the dataset into a training and validation set, as well as a test set, with a ratio of 9:1; Kinetics-Sounds (*40*), contains 31 human action categories selected from the Kinetics dataset (*73*). The dataset contains 17,366 10-s video clips, of which 1472 are training and validation samples and 2594 are test samples; UrbanSound8K-AV dataset (*41*), with 8732 audiovisual samples totaling 10 categories. Each sample consists of a color image and a 4-s audio signal. We randomly divided the dataset into training and test sets in the ratio of 7:3. Audiovisual continual learning: We used the class-incremental audiovisual dataset introduced by Pian *et al.* (*49*), comprising three benchmark datasets: AVE-CI (four tasks × seven classes) consisting of 3294 training samples, 391 validation samples, and 394 test samples; K-S-CI (five tasks × six classes) containing 19,220 training samples, 1947 validation samples, and 1958 test samples; and VS100-CI (10 tasks × 10 classes) with 51,195 training samples, 5000 validation samples, and 5000 test samples. For AVQA, we used the official MUSIC-AVQA (*50*) split, which contains 32,087 training, 4595 validation, and 9185 test question-answer pairs. For VL tasks, we used the Hateful Memes dataset (*42*), a multimodal dataset for hate speech detection containing 10,000 image-text pairs. We followed the official “seen” splits for training, validation, and testing. For trimodal tasks, we used the MELD dataset (*45*) for emotion recognition, which contains 13,708 utterances from the TV series *Friends* split into 9989 training, 1109 validation, and 2610 testing utterances. For sentiment analysis, we used the CMU-MOSI dataset (*46*), consisting of 2199 opinion video segments divided into 1284 training, 229 validation, and 686 testing samples.

#### Data processing and network backbones

All raw videos were first resampled to a uniform frame rate. According to different task settings, we randomly sampled 1, 3, or 16 frames from each video clip as the visual input. The corresponding audio input was transformed into log-Mel spectrograms, which were used as input to the audio branch. For audiovisual classification, we used the ResNet-18 (*74*) architecture for both the visual and audio streams. To investigate architectural generality, we adapted this topology to an SNN counterpart by replacing conventional activation functions with LIF neurons; neuron-level hyperparameters are detailed in table S1. For audiovisual continual learning, we used VideoMAE (*75*) and AudioMAE (*76*) to extract video frames and audio features. For audiovisual question and answer, for vision, we used pretrained ResNet-18 model, and for audio, we used pretrained VGGish (*77*) to extract visual and audio features respectively. For VL experiments, we evaluated three architectures: Concat BERT (*42*), where we replaced the original ResNet-152 visual backbone with a vision transformer, ViT-B/16 (*78*) to concatenate with BERT (*79*) text features; MMBT-Grid (*43*), which projects visual features into the text token space; and ViLBERT (*44*), which uses a dual-stream co-attention transformer mechanism. To extend beyond pairwise fusion, we conducted a three-modality experiment on the MELD dataset. We used preextracted ResNet101 (*74*) visual features, Wav2Vec2.0 (*80*) audio features, and GloVe-initialized text embeddings (*81*). The three modalities were projected to a unified space, concatenated, and processed by a bidirectional LSTM for dialogue-level emotion recognition. For sentiment analysis on the CMU-MOSI dataset, we adopted the Multimodal-InfoMax framework (*82*), which synthesizes fusion results through hierarchical mutual information maximization.

#### Optimization details

Optimization strategies were tailored to different task domains to accommodate specific architectural requirements.

1. Audiovisual tasks: For audiovisual classification, we used stochastic gradient descent (SGD) (*83*) with a weight decay of $1\times 10^{-4}$, training for 100 epochs with an initial learning rate of $5\times 10^{-3}$ and a batch size of 32. In the audiovisual continual learning task, models were trained for 100 epochs with a learning rate of $1\times 10^{-2}$ and a batch size of 256; notably, for the VS100-CI benchmark, we used the Adam optimizer (*70*) ($\beta_{1}=0.9$, $\beta_{2}=0.999$) to handle gradient noise. AVQA models were trained for 50 epochs using SGD with a learning rate of $1\times 10^{-2}$ and a batch size of 64.

2. VL tasks: For the Hateful Memes dataset, we used the SGD optimizer configured with a learning rate of $7\times 10^{-5}$, a momentum of 0.9, and a weight decay of $1\times 10^{-4}$. The models were trained for 50 epochs to ensure convergence on the multimodal objective.

3. Trimodal tasks: For tasks involving text, audio, and visual modalities, we adopted the Adam optimizer. On the MELD emotion recognition dataset, we set the learning rate to $1\times 10^{-4}$ and weight decay to $1\times 10^{-5}$, training for 50 epochs. For sentiment analysis on CMU-MOSI, the model was trained for 40 epochs using Adam with a weight decay of $1\times 10^{-4}$.

#### Training configuration and hyperparameters

Table S1 details the hyperparameter settings used across the diverse multimodal tasks reported in the Results. To ensure fair comparison with baselines and demonstrate the robustness of the proposed IEMF method, we adopted the following selection protocol:

1. Optimization and backbone settings: For general training parameters, including optimizer, learning rate, weight decay, and batch size, we followed standard protocols established in the respective benchmark literature. This ensures that any performance improvement is attributed to the IEMF mechanism rather than superior backbone tuning.

2. IEMF parameter configuration: We deliberately avoided extensive hyperparameter search to validate the method’s generalization capability.

(a) Initial selection: The inverse effectiveness coefficient γ was determined via a lightweight preliminary evaluation on the validation split of the audiovisual classification task, where γ=1.0 yielded stable performance.

(b) Robustness verification: Crucially, this value (γ=1.0) and the gating function κ(⋅)=tanh(⋅) were kept fixed for all subsequent experiments (including continual learning, QA, VL, and trimodal tasks). The consistent gains confirm that IEMF is architecture-agnostic and does not rely on task-specific fine-tuning.

#### Experimental platform

All experiments were conducted on a linux server equipped with NVIDIA A100-40 GB GPUs and an AMD EPYC 7763 processor.

### Details of evaluation metrics

We evaluate the proposed method across three multimodal tasks using task-specific metrics.

1.Audiovisual classification: For audiovisual classification tasks, we report the standard Top-1 accuracy, $\mathrm{Acc}=\frac{1}{N}\sum_{i=1}^{N}1\left({\hat{y}}_{i}=y_{i}\right)$, where $1\left({\hat{y}}_{i}=y_{i}\right)$ represents the indicator function, which equals 1 if ${\hat{y}}_{i}=y_{i}$ and 0 otherwise. This metric measures the proportion of examples where the predicted label matches the ground truth. To further assess the computational cost during training, inspired by Zhang *et al.* (*84*), we fairly compare the efficiency of different methods by considering both the number of epochs required to reach specified error rates and the computational complexity per epoch. Formally, the computational cost for an algorithm is defined as $\text{Cost}=\frac{1}{L}\sum_{l=1}^{L}\text{Argmin}\left[f\left(x\right)\leq {\mathrm{Err}}_{l}\right]\times \Omega_{e}.$ In this formula, L represents the number of predefined error rate thresholds (set to 5 in our experiments, ${\mathrm{Err}}_{l}$ denotes the predefined error rate levels, $\text{Argmin}\left[f\left(x\right)\leq {\mathrm{Err}}_{l}\right]$ is the first epoch at which the algorithm reaches or goes below the specified error rate ${\mathrm{Err}}_{l}$, and $\Omega_{e}$ represents the algorithmic complexity per epoch, measured in FLOPs. We define the error rate ${\mathrm{Err}}_{l}$ using upper and lower bounds determined from the training curves of all methods under comparison. Specifically, the upper bound is set as the minimum value among the highest error rates of all compared methods. The lower bound is set to the maximum of the lowest values of the final error rates of the various methods. Within this range, we choose error rate thresholds at uniform intervals to ensure that the entire performance interval has been systematically and fairly evaluated.

2.Audiovisual continual learning. We track model accuracy throughout the training process using two metrics: average accuracy (AA) and average incremental accuracy (AIA).

At each step k (i.e., after learning the kth task), we compute the average accuracy ${\text{AA}}_{k}$ across all tasks encountered so far as ${\text{AA}}_{k}=\frac{1}{k}\sum_{j=1}^{k}a_{k,j}$, where $a_{k,j}$ is the accuracy on the *j*-th task after learning task k, and j≤k. To summarize performance across the entire learning sequence, we report the average incremental accuracy, which is the mean of AA values over all *K* tasks $\text{AIA}=\frac{1}{K}\sum_{i=1}^{K}{\text{AA}}_{i}$. Here, AA reflects the model’s performance after each task, while AIA captures the overall trend and stability of learning across all tasks.

To further quantify how much the model forgets previous tasks, we introduce the average forgetting rate (AFR) in the appendix table S5. Let $a_{k,j}$ be the test accuracy on task j after learning task k. The forgetting on task k is defined as $F_{k}=\frac{1}{k-1}\sum_{j=1}^{k-1}{\text{max}}_{1\leq \ell \leq k-1}\left(a_{\ell ,j}-a_{k,j}\right)$, i.e., the average drop from the highest accuracy ever achieved on task *j* to its accuracy after the final task. For a total of *K* tasks, then $\text{AFR}=\frac{1}{K-1}\sum_{k=2}^{K}F_{k}$, where $F_{k}$ excludes the first task (as forgetting can only be measured after learning at least two tasks). This metric summarizes how much the model’s performance on previously learned tasks degrades over the entire learning sequence.

3. AVQA. We evaluate modal-specific question and answer accuracy, denoted by $\left(A_{a},A_{v},A_{\mathrm{av}}\right)$, which respectively evaluate performance across audio-only, visual-only, and audiovisual questions. Each accuracy is computed as $\frac{1}{N}\sum_{i=1}^{N}1\left({\hat{\text{ans}}}_{i}={\text{ans}}_{i}\right),$ which measures exact match between predicted and ground-truth answers over all question types within each modality.

4. VL and trimodal evaluation. For VL tasks (e.g., Hateful Memes), we report the AUROC and F1-Macro score. The AUROC quantifies the discriminative capability of the model across various threshold settings $\text{AUROC}=\int_{0}^{1}\text{TPR}\left[{\text{FPR}}^{-1}\left(t\right)\right]\;\mathrm{dt},$ where TPR is the true-positive rate and FPR is the false-positive rate. The F1-Macro score is calculated as the unweighted mean of the F1 scores for each class, ensuring equal contribution from all classes regardless of their frequency.

For trimodal tasks (e.g., MELD and CMU-MOSI), to account for class imbalance, we use the weighted-F1 and F1 score. The F1 score is the harmonic mean of precision (P) and recall (R): $F1=2\cdot \frac{P\cdot R}{P+R}.$ For sentiment analysis on the CMU-MOSI dataset, we report the binary classification accuracy (Acc-2). Following standard protocol (*46*), this metric evaluates the model’s ability to classify samples into negative and nonnegative (including neutral and positive) categories. It is defined as $\text{Acc}-2=\frac{N_{\text{correct}}}{N_{\text{total}}}=\frac{\sum_{i=1}^{N}I\left[\left({\hat{y}}_{i}\geq 0\right)=\left(y_{i}\geq 0\right)\right]}{N}$ where ${\hat{y}}_{i}$ and $y_{i}$ are the predicted and ground-truth sentiment scores, respectively, and I(⋅) is the indicator function.

## Supplementary Materials

### This PDF file includes:

Algorithm S1 Tables S1 to S11

## References

[1] M. O. Ernst, H. H. Bülthoff, Merging the senses into a robust percept. Trends Cogn. Sci. 8, 162–169 (2004). 15050512 10.1016/j.tics.2004.02.002

[2] U. Noppeney, Perceptual inference, learning, and attention in a multisensory world. Annu. Rev. Neurosci. 44, 449–473 (2021). 33882258 10.1146/annurev-neuro-100120-085519

[3] A. Nagrani, S. Yang, A. Arnab, A. Jansen, C. Schmid, C. Sun, Attention bottlenecks for multimodal fusion. Adv. Neural Inf. Proces. Syst. 34, 14200–14213 (2021).

[4] X. Peng, Y. Wei, A. Deng, D. Wang, D. Hu, “Balanced multimodal learning via on-the-fly gradient modulation” in *Proceedings of the IEEE/CVF Conference on Computer Vision and Pattern Recognition* (2022), pp. 8238–8247.

[5] F. Yu, Y. Wu, S. Ma, M. Xu, H. Li, H. Qu, C. Song, T. Wang, R. Zhao, L. Shi, Brain-inspired multimodal hybrid neural network for robot place recognition. Sci. Robot. 8, eabm6996 (2023). 37163608 10.1126/scirobotics.abm6996

[6] R. Jiang, J. Han, Y. Xue, P. Wang, H. Tang, “CMCI: A robust multimodal fusion method for spiking neural networks” in *International Conference on Neural Information Processing* (Springer, 2024), pp. 159–171.

[7] M. U. K. Sadaf, N. U. Sakib, A. Pannone, H. Ravichandran, S. Das, A bio-inspired visuotactile neuron for multisensory integration. Nat. Commun. 14, 5729 (2023). 37714853 10.1038/s41467-023-40686-z PMC10504285

[8] F. Lv, X. Chen, Y. Huang, L. Duan, G. Lin, “Progressive modality reinforcement for human multimodal emotion recognition from unaligned multimodal sequences” in *Proceedings of the IEEE/CVF Conference on Computer Vision and Pattern Recognition* (2021), pp. 2554–2562.

[9] Z. Cheng, Z.-Q. Cheng, J.-Y. He, K. Wang, Y. Lin, Z. Lian, X. Peng, A. Hauptmann, Emotion-llama: Multimodal emotion recognition and reasoning with instruction tuning. Adv. Neural Inf. Proces. Syst. 37, 110805–110853 (2024).

[10] Y. Mroueh, E. Marcheret, V. Goel, “Deep multimodal learning for audio-visual speech recognition” in *2015 IEEE International Conference on Acoustics, Speech and Signal Processing (ICASSP)* (IEEE, 2015), pp. 2130–2134.

[11] M. Kim, J. Hong, S. J. Park, Y. M. Ro, Cromm-vsr: Cross-modal memory augmented visual speech recognition. IEEE Trans. Multimed. 24, 4342–4355 (2021).

[12] J. H. Yeo, M. Kim, J. Choi, D. H. Kim, Y. M. Ro, Akvsr: Audio knowledge empowered visual speech recognition by compressing audio knowledge of a pretrained model. IEEE Trans. Multimed. 26, 6462–6474 (2024).

[13] A. Radford, J. W. Kim, C. Hallacy, A. Ramesh, G. Goh, S. Agarwal, G. Sastry, A. Askell, P. Mishkin, J. Clark, G. Krueger, I. Sutskever, “Learning transferable visual models from natural language supervision” in *Proceedings of the 38th International Conference on Machine Learning* (PMLR, 2021), vol. 139 of *Proceedings of Machine Learning Research*, pp. 8748–8763.

[14] C. Jia, Y. Yang, Y. Xia, Y.-T. Chen, Z. Parekh, H. Pham, Q. Le, Y.-H. Sung, Z. Li, T. Duerig, “Scaling up visual and vision-language representation learning with noisy text supervision” in *International Conference on Machine Learning* (PMLR, 2021), pp. 4904–4916.

[15] X. Zhai, X. Wang, B. Mustafa, A. Steiner, D. Keysers, A. Kolesnikov, L. Beyer, “Lit: Zero-shot transfer with locked-image text tuning” in *Proceedings of the IEEE/CVF Conference on Computer Vision and Pattern Recognition* (2022), pp. 18123–18133.

[16] H. Tan, M. Bansal, LXMERT: Learning cross-modality encoder representations from transformers. arXiv:1908.07490 [cs.CL] (2019).

[17] Y.-H. H. Tsai, S. Bai, P. P. Liang, J. Z. Kolter, L.-P. Morency, R. Salakhutdinov, “Multimodal transformer for unaligned multimodal language sequences” in *Proceedings of the Conference. Association for Computational Linguistics. Meeting* (2019), vol. 2019, p. 6558. 10.18653/v1/p19-1656 PMC7195022 32362720

[18] X. Li, X. Yin, C. Li, P. Zhang, X. Hu, L. Zhang, L. Wang, H. Hu, L. Dong, F. Wei, Y. Choi, J. Gao, “Oscar: Object-semantics aligned pre-training for vision-language tasks” in *European Conference on Computer Vision* (Springer, 2020), pp. 121–137.

[19] Y. Yao, R. Mihalcea, “Modality-specific learning rates for effective multimodal additive late-fusion” in *Findings of the Association for Computational Linguistics: ACL 2022* (2022), pp. 1824–1834.

[20] Y. Yang, F. Wan, Q.-Y. Jiang, Y. Xu, Facilitating multimodal classification via dynamically learning modality gap. Adv. Neural Inf. Proces. Syst. 37, 62108–62122 (2024).

[21] A. Kendall, Y. Gal, What uncertainties do we need in Bayesian deep learning for computer vision? Adv. Neural Inf. Proces. Syst. 30, 5574–5584 (2017).

[22] Z. Han, F. Yang, J. Huang, C. Zhang, J. Yao, “Multimodal dynamics: Dynamical fusion for trustworthy multimodal classification” in *Proceedings of the IEEE/CVF Conference on Computer Vision and Pattern Recognition* (2022), pp. 20707–20717.

[23] C. Qian, K. Han, J. Wang, Z. Yuan, C. Lyu, J. Chen, Z. Liu, Dyncim: Dynamic curriculum for imbalanced multimodal learning*.* arXiv:2503.06456 [cs.CV] (2025).

[24] D. A. Bulkin, J. M. Groh, Seeing sounds: Visual and auditory interactions in the brain. Curr. Opin. Neurobiol. 16, 415–419 (2006). 16837186 10.1016/j.conb.2006.06.008

[25] J. Enoch, L. McDonald, L. Jones, P. R. Jones, D. P. Crabb, Evaluating whether sight is the most valued sense. JAMA Ophthalmol. 137, 1317–1320 (2019). 31580383 10.1001/jamaophthalmol.2019.3537 PMC6777262

[26] E. Macaluso, J. Driver, Multisensory spatial interactions: A window onto functional integration in the human brain. Trends Neurosci. 28, 264–271 (2005). 15866201 10.1016/j.tins.2005.03.008

[27] G. A. Calvert, R. Campbell, M. J. Brammer, Evidence from functional magnetic resonance imaging of crossmodal binding in the human heteromodal cortex. Curr. Biol. 10, 649–657 (2000). 10837246 10.1016/s0960-9822(00)00513-3

[28] E. Macaluso, N. George, R. Dolan, C. Spence, J. Driver, Spatial and temporal factors during processing of audiovisual speech: A PET study. Neuroimage 21, 725–732 (2004). 14980575 10.1016/j.neuroimage.2003.09.049

[29] T. Noesselt, J. W. Rieger, M. A. Schoenfeld, M. Kanowski, H. Hinrichs, H.-J. Heinze, J. Driver, Audiovisual temporal correspondence modulates human multisensory superior temporal sulcus plus primary sensory cortices. J. Neurosci. 27, 11431–11441 (2007). 17942738 10.1523/JNEUROSCI.2252-07.2007 PMC2957075

[30] N. Van Atteveldt, E. Formisano, R. Goebel, L. Blomert, Integration of letters and speech sounds in the human brain. Neuron 43, 271–282 (2004). 15260962 10.1016/j.neuron.2004.06.025

[31] D. Senkowski, D. Saint-Amour, T. Gruber, J. J. Foxe, Look who’s talking: The deployment of visuo-spatial attention during multisensory speech processing under noisy environmental conditions. Neuroimage 43, 379–387 (2008). 18678262 10.1016/j.neuroimage.2008.06.046 PMC2596295

[32] G. R. Szycik, P. Tausche, T. F. Münte, A novel approach to study audiovisual integration in speech perception: Localizer fMRI and sparse sampling. Brain Res. 1220, 142–149 (2008). 17880929 10.1016/j.brainres.2007.08.027

[33] M. Avillac, S. Deneve, E. Olivier, A. Pouget, J.-R. Duhamel, Reference frames for representing visual and tactile locations in parietal cortex. Nat. Neurosci. 8, 941–949 (2005). 15951810 10.1038/nn1480

[34] C. Regenbogen, J. Seubert, E. Johansson, A. Finkelmeyer, P. Andersson, J. N. Lundström, The intraparietal sulcus governs multisensory integration of audiovisual information based on task difficulty. Hum. Brain Mapp. 39, 1313–1326 (2018). 29235185 10.1002/hbm.23918 PMC6866436

[35] K. O. Bushara, J. Grafman, M. Hallett, Neural correlates of auditory–visual stimulus onset asynchrony detection. J. Neurosci. 21, 300–304 (2001). 11150347 10.1523/JNEUROSCI.21-01-00300.2001 PMC6762435

[36] N. E. Barraclough, D. Xiao, C. I. Baker, M. W. Oram, D. I. Perrett, Integration of visual and auditory information by superior temporal sulcus neurons responsive to the sight of actions. J. Cogn. Neurosci. 17, 377–391 (2005). 15813999 10.1162/0898929053279586

[37] B. E. Stein, T. R. Stanford, Multisensory integration: Current issues from the perspective of the single neuron. Nat. Rev. Neurosci. 9, 255–266 (2008). 18354398 10.1038/nrn2331

[38] C. R. Fetsch, G. C. DeAngelis, D. E. Angelaki, Bridging the gap between theories of sensory cue integration and the physiology of multisensory neurons. Nat. Rev. Neurosci. 14, 429–442 (2013). 23686172 10.1038/nrn3503 PMC3820118

[39] H. Cao, D. G. Cooper, M. K. Keutmann, R. C. Gur, A. Nenkova, R. Verma, Crema-d: Crowd-sourced emotional multimodal actors dataset. IEEE Trans. Affect. Comput. 5, 377–390 (2014). 25653738 10.1109/TAFFC.2014.2336244 PMC4313618

[40] R. Arandjelovic, A. Zisserman, “Look, listen, and learn” in *Proceedings of the IEEE International Conference on Computer Vision* (2017), pp. 609–617.

[41] L. Guo, Z. Gao, J. Qu, S. Zheng, R. Jiang, Y. Lu, H. Qiao, Transformer-based spiking neural networks for multimodal audio-visual classification. IEEE Trans. Cogn. Dev. Syst. 16, 1077–1086 (2024).

[42] D. Kiela, H. Firooz, A. Mohan, V. Goswami, A. Singh, P. Ringshia, D. Testuggine, The hateful memes challenge: Detecting hate speech in multimodal memes. Adv. Neural Inf. Proces. Syst. 33, 2611–2624 (2020).

[43] D. Kiela, S. Bhooshan, H. Firooz, E. Perez, D. Testuggine, Supervised multimodal bitransformers for classifying images and text. arXiv:1909.02950 [cs.CL] (2019).

[44] J. Lu, D. Batra, D. Parikh, S. Lee, Vilbert: Pretraining task-agnostic visiolinguistic representations for vision-and-language tasks. Adv. Neural Inf. Proces. Syst. 32, (2019).

[45] S. Poria, D. Hazarika, N. Majumder, G. Naik, E. Cambria, R. Mihalcea, “Meld: A multimodal multi-party dataset for emotion recognition in conversations” in *Proceedings of the 57th Annual Meeting of the Association for Computational Linguistics* (2019), pp. 527–536.

[46] A. Zadeh, R. Zellers, E. Pincus, L.-P. Morency, Multimodal sentiment intensity analysis in videos: Facial gestures and verbal messages. IEEE Intell. Syst. 31, 82–88 (2016).

[47] Z. Li, D. Hoiem, Learning without forgetting. IEEE Trans. Pattern Anal. Mach. Intell. 40, 2935–2947 (2018). 29990101 10.1109/TPAMI.2017.2773081

[48] H. Ahn, J. Kwak, S. Lim, H. Bang, H. Kim, T. Moon, “Ss-il: Separated softmax for incremental learning” in *Proceedings of the IEEE/CVF International Conference on Computer Vision* (2021), pp. 844–853.

[49] W. Pian, S. Mo, Y. Guo, Y. Tian, “Audio-visual class-incremental learning” in *Proceedings of the IEEE/CVF International Conference on Computer Vision* (2023), pp. 7799–7811.

[50] G. Li, Y. Wei, Y. Tian, C. Xu, J.-R. Wen, D. Hu, “Learning to answer questions in dynamic audio-visual scenarios” in *Proceedings of the IEEE/CVF Conference on Computer Vision and Pattern Recognition* (2022), pp. 19108–19118.

[51] X. He, D. Zhao, Y. Dong, G. Shen, X. Yang, Y. Zeng, Enhancing audio-visual spiking neural networks through semantic-alignment and cross-modal residual learning. arXiv:2502.12488 [cs.CV] (2025).

[52] B. E. Stein, T. R. Stanford, B. A. Rowland, Development of multisensory integration from the perspective of the individual neuron. Nat. Rev. Neurosci. 15, 520–535 (2014). 25158358 10.1038/nrn3742 PMC4215474

[53] D. A. Slutsky, G. H. Recanzone, Temporal and spatial dependency of the ventriloquism effect. Neuroreport 12, 7–10 (2001). 11201094 10.1097/00001756-200101220-00009

[54] W. D. Hairston, D. A. Hodges, J. H. Burdette, M. T. Wallace, Auditory enhancement of visual temporal order judgment. Neuroreport 17, 791–795 (2006). 16708016 10.1097/01.wnr.0000220141.29413.b4

[55] B. E. Stein, M. A. Meredith, *The Merging of the Senses* (MIT Press, 1993).

[56] C. Liu, S. Ding, H. J. Kim, S. Long, D. Xiao, S. Ghazanfar, P. Yang, Multitask benchmarking of single-cell multimodal omics integration methods. Nat. Methods 22, 2449–2460 (2025). 41083898 10.1038/s41592-025-02856-3 PMC12615258

[57] V. Svensson, Droplet scRNA-seq is not zero-inflated. Nat. Biotechnol. 38, 147–150 (2020). 31937974 10.1038/s41587-019-0379-5

[58] C. Xiao, Y. Chen, Q. Meng, L. Wei, X. Zhang, Benchmarking multi-omics integration algorithms across single-cell RNA and ATAC data. Brief. Bioinform. 25, bbae095 (2024). 38493343 10.1093/bib/bbae095 PMC10944570

[59] Z.-J. Cao, G. Gao, Multi-omics single-cell data integration and regulatory inference with graph-linked embedding. Nat. Biotechnol. 40, 1458–1466 (2022). 35501393 10.1038/s41587-022-01284-4 PMC9546775

[60] T. Ashuach, M. I. Gabitto, R. V. Koodli, G.-A. Saldi, M. I. Jordan, N. Yosef, MultiVI: Deep generative model for the integration of multimodal data. Nat. Methods 20, 1222–1231 (2023). 37386189 10.1038/s41592-023-01909-9 PMC10406609

[61] M. Xu, L. Cai, Z. Yang, R. Wang, H. Zhang, Gene-guided multimodal data fusion for cancer patient survival analysis. Neurocomputing 668, 132365 (2026).

[62] F. Gao, J. Ding, B. Gai, D. Cai, C. Hu, F.-A. Wang, R. He, J. Liu, Y. Li, X.-J. Wu, Interpretable multimodal fusion model for bridged histology and genomics survival prediction in pan-cancer. Adv. Sci. 12, e2407060 (2025). 10.1002/advs.202407060 PMC12061278 40051298

[63] Z. Liu, Y. Wu, H. Xu, M. Wang, S. Weng, D. Pei, S. Chen, W. Wang, J. Yan, L. Cui, J. Duan, Y. Zhao, Z. Wang, Z. Ma, R. Li, W. Duan, Y. Qiu, D. Su, S. Li, H. Liu, W. Li, C. Ma, M. Yu, Y. Yu, T. Chen, J. Fu, Y. Zhen, B. Yu, Y. Ji, H. Zheng, D. Liang, X. Liu, D. Yan, X. Han, F. Wang, Z.-C. Li, Z. Zhang, Multimodal fusion of radio-pathology and proteogenomics identify integrated glioma subtypes with prognostic and therapeutic opportunities. Nat. Commun. 16, 3510 (2025). 40222975 10.1038/s41467-025-58675-9 PMC11994800

[64] N. K. Logothetis, J. Pauls, M. Augath, T. Trinath, A. Oeltermann, Neurophysiological investigation of the basis of the fMRI signal. Nature 412, 150–157 (2001). 11449264 10.1038/35084005

[65] J. Sui, T. Adali, Q. Yu, J. Chen, V. D. Calhoun, A review of multivariate methods for multimodal fusion of brain imaging data. J. Neurosci. Methods 204, 68–81 (2012). 22108139 10.1016/j.jneumeth.2011.10.031 PMC3690333

[66] Z. Lin, H. Akin, R. Rao, B. Hie, Z. Zhu, W. Lu, N. Smetanin, R. Verkuil, O. Kabeli, Y. Shmueli, A. D. S. Costa, M. Fazel-Zarandi, T. Sercu, S. Candido, A. Rives, Evolutionary-scale prediction of atomic-level protein structure with a language model. Science 379, 1123–1130 (2023). 36927031 10.1126/science.ade2574

[67] J. Su, C. Han, Y. Zhou, J. Shan, X. Zhou, F. Yuan, “SaProt: Protein language modeling with structure-aware vocabulary” in *The Twelfth International Conference on Learning Representations* (2024).

[68] V. Nair, G. E. Hinton, “Rectified linear units improve restricted boltzmann machines” in *Proceedings of the 27th International Conference on Machine Learning (ICML-10)* (2010), pp. 807–814.

[69] P. Dayan, L. F. Abbott, *Theoretical Neuroscience: Computational and Mathematical Modeling of Neural Systems* (MIT Press, 2005).

[70] D. P. Kingma, J. Ba, “Adam: A method for stochastic optimization” in *3rd International Conference on Learning Representations, ICLR 2015* (2015).

[71] N. S. Keskar, J. Nocedal, P. T. P. Tang, D. Mudigere, M. Smelyanskiy, “On large-batch training for deep learning: Generalization gap and sharp minima” in *5th International Conference on Learning Representations, ICLR 2017* (2017).

[72] P. Foret, A. Kleiner, H. Mobahi, B. Neyshabur, “Sharpness-aware minimization for efficiently improving generalization,” in *9th International Conference on Learning Representations* (2021).

[73] W. Kay, J. Carreira, K. Simonyan, B. Zhang, C. Hillier, S. Vijayanarasimhan, F. Viola, T. Green, T. Back, P. Natsev, M. Suleyman, A. Zisserman, The kinetics human action video dataset. arXiv:1705.06950 [cs.CV] (2017).

[74] K. He, X. Zhang, S. Ren, J. Sun, “Deep residual learning for image recognition” in *Proceedings of the IEEE Conference on Computer Vision and Pattern Recognition* (2016), pp. 770–778.

[75] Z. Tong, Y. Song, J. Wang, L. Wang, Videomae: Masked autoencoders are data-efficient learners for self-supervised video pre-training. Adv. Neural Inf. Proces. Syst. 35, 10078–10093 (2022).

[76] P.-Y. Huang, H. Xu, J. Li, A. Baevski, M. Auli, W. Galuba, F. Metze, C. Feichtenhofer, Masked autoencoders that listen. Adv. Neural Inf. Proces. Syst. 35, 28708–28720 (2022).

[77] S. Hershey, S. Chaudhuri, D. P. W. Ellis, J. F. Gemmeke, A. Jansen, R. C. Moore, M. Plakal, D. Platt, R. A. Saurous, B. Seybold, M. Slaney, R. J. Weiss, K. Wilson, “CNN architectures for large-scale audio classification” in *2017 IEEE International Conference on Acoustics, Speech and Signal Processing (ICASSP)* (IEEE, 2017), pp. 131–135.

[78] A. Dosovitskiy, An image is worth 16x16 words: Transformers for image recognition at scale. arXiv:2010.11929 [cs.CV] (2020).

[79] J. Devlin, Bert: Pre-training of deep bidirectional transformers for language understanding. arXiv:1810.04805 [cs.CL] (2018).

[80] A. Baevski, Y. Zhou, A. Mohamed, M. Auli, wav2vec 2.0: A framework for self-supervised learning of speech representations. Adv. Neural Inf. Process. Syst. 33, 12449–12460 (2020).

[81] J. Pennington, R. Socher, C. D. Manning, “Glove: Global vectors for word representation” in *Proceedings of the 2014 Conference on Empirical Methods in Natural Language Processing (EMNLP)* (2014), pp. 1532–1543.

[82] W. Han, H. Chen, S. Poria, Improving multimodal fusion with hierarchical mutual information maximization for multimodal sentiment analysis. arXiv:2109.00412 [cs.CL] (2021).

[83] H. Robbins, S. Monro, A stochastic approximation method. Ann. Math. Stat. 22, 400–407 (1951).

[84] T. Zhang, X. Cheng, S. Jia, M. Poo, Y. Zeng, B. Xu, Self-backpropagation of synaptic modifications elevates the efficiency of spiking and artificial neural networks. Sci. Adv. 7, eabh0146 (2021). 34669481 10.1126/sciadv.abh0146 PMC8528419

[85] A. Paszke, S. Gross, F. Massa, A. Lerer, J. Bradbury, G. Chanan, T. Killeen, Z. Lin, N. Gimelshein, L. Antiga, A. Desmaison, A. Kopf, E. Yang, Z. DeVito, M. Raison, A. Tejani, S. Chilamkurthy, B. Steiner, L. Fang, J. Bai, S. Chintala, “PyTorch: An imperative style, high-performance deep learning library” in *Advances in Neural Information Processing Systems*, H. Wallach, H. Larochelle, A. Beygelzimer, F. d’Alché-Buc, E. Fox, R. Garnett, Eds. (NeurIPS, 2019).

