# Supplementary material for: Incorporating brain-inspired mechanisms for multimodal learning in artificial intelligence
Source: Sci Adv. 2026 Jun 5;12(23):eady8751. doi: 10.1126/sciadv.ady8751 (PMC13240175; doi:10.1126/sciadv.ady8751)
Supplement: Supplementary file 1 — Algorithm S1 Tables S1 to S11 [file sciadv.ady8751_sm.pdf]

Science Advances
AAAS

Supplementary Materials for

Incorporating brain-inspired mechanisms for multimodal learning in artificial intelligence

Xiang He *et al.*

Corresponding author: Qingqun Kong, qingqun.kong@ia.ac.cn; Xin Yang, xin.yang@ia.ac.cn; Yi Zeng, yi.zeng@ia.ac.cn

Sci. Adv. **12**, eady8751 (2026)
DOI: 10.1126/sciadv.ady8751

This PDF file includes:

Algorithm S1
Tables S1 to S11

Algorithm 1

Algorithm 1: Pseudocode of the proposed Inverse Effectiveness Driven Multimodal Fusion (IEMF) algorithm.

Input: Multimodal dataset $\mathcal{D}$, Encoders φ^a, φ^v, Fusion module $\mathcal{F}$ (params $\mathbf{W}^f$), Classifier h, Unimodal heads parameters $\mathbf{W}^{a/v}$, $\mathbf{b}^{a/v}$, Learning rate η, Gain coefficient γ.

Output: Optimized model parameters $\boldsymbol{\theta}$. *(Note: During inference, the optimized parameters are used for standard forward pass without IEMF modulation.)*

for *each training epoch* **do**

  for *each mini-batch* $\mathcal{B}_t = \{(\boldsymbol{x}_i^a, \boldsymbol{x}_i^v, y_i)\}_{i=1}^{|\mathcal{B}_t|}$ *sampled from* $\mathcal{D}$ **do**

    `// Phase 1: Forward Pass & Feature Extraction`

    Obtain latent features: $\boldsymbol{z}_i^a = \varphi^a(\boldsymbol{x}_i^a)$, $\boldsymbol{z}_i^v = \varphi^v(\boldsymbol{x}_i^v)$

    Obtain fused representation: $\boldsymbol{z}_i^{\mathrm{av}} = \mathcal{F}(\boldsymbol{z}_i^a, \boldsymbol{z}_i^v)$

    Compute prediction: $\hat{\boldsymbol{y}}_i = h(\boldsymbol{z}_i^{\mathrm{av}})$

    `// Phase 2: Informativeness Evaluation (Eq. 7, 8)`

    Compute probabilities $\mathbf{p}_i^a, \mathbf{p}_i^v, \mathbf{p}_i^{\mathrm{av}}$.

    Extract information content based on ground-truth y_i:

      $c_i^a \leftarrow [\mathbf{p}_i^a]_{y_i}, \quad c_i^v \leftarrow [\mathbf{p}_i^v]_{y_i}, \quad c_i^{\mathrm{av}} \leftarrow [\mathbf{p}_i^{\mathrm{av}}]_{y_i}$

    `// Phase 3: IEMF Coefficient Calculation`

    Calculate batch-level scores (Eq. 6):

      $S_t^{a-v} \leftarrow \frac{1}{2|\mathcal{B}_t|}\sum(c_i^a + c_i^v), \quad S_t^{av} \leftarrow \frac{1}{|\mathcal{B}_t|}\sum c_i^{av}$

    Compute fusion coefficient ξ_t (Eq. 10):

      $\xi_t \leftarrow \gamma \cdot \left(1 + \tanh\left(1 - \frac{S_t^{a-v}}{S_t^{av} + \epsilon}\right)\right), \quad \epsilon = 10^{-8}$ `// ϵ for numerical stability`

    `// Phase 4: Backpropagation & Parameter Update`

    Compute task loss: $\mathcal{L}(\boldsymbol{\theta}) = \frac{1}{|\mathcal{B}_t|}\sum_i \mathcal{L}_{\mathrm{ce}}(\hat{\boldsymbol{y}}_i, y_i)$

    Compute gradients: $\nabla_{\boldsymbol{\theta}}\mathcal{L}$

    Update non-fusion params (standard SGD/Adam):

      $\boldsymbol{\theta}^{\setminus f} \leftarrow \boldsymbol{\theta}^{\setminus f} - \eta \cdot \nabla_{\boldsymbol{\theta}^{\setminus f}}\mathcal{L}$

    Update fusion params with **IEMF modulation** (Eq. 11):

      $\mathbf{W}_{t+1}^f \leftarrow \mathbf{W}_t^f - \eta \cdot \xi_t \cdot \nabla_{\mathbf{W}^f}\mathcal{L}$

Table S1.

Parameters for diverse multimodal benchmark tasks.

Category	Parameters	Values
Audio Visual Classification	Network backbone	ResNet-18
	Optimizer	SGD
	Weight decay	1×10^{-4}
	Initial learning rate	5×10^{-3}
	Number of training epochs	100
	Batch size	32
LIF Neuron (SNN)	Resting potential V_{rest}	0
	Firing threshold V_{th}	0.5
	Membrane time constant τ_m	2.0
	Surrogate Gradient Function	Piecewise linear
	Conductivity g_L, g_E, g_I	1 S, 1 S, 1 S
	Reversal potential V_E, V_I	0
	Discrete time step t	4
Audio Visual Continual Learning	Network backbone	VideoMAE (Visual), AudioMAE (Audio)
	Learning rate	1×10^{-2}
	Batch size	256
	Number of training epochs	100
	Optimizer	SGD (Default) / Adam (VS100-CI)
Audio Visual Question and Answering	Network backbone	ResNet-18 (Visual), VGGish (Audio)
	Optimizer	SGD
	Learning rate	1×10^{-2}
	Batch size	64
	Number of training epochs	50
Vision-Language Tasks	Network backbone	ViT-B/16 + BERT / ViLBERT
	Optimizer	SGD (Momentum 0.9)
	Learning rate	7×10^{-5}
	Weight decay	1×10^{-4}
	Number of training epochs	50
Tri-modal Tasks	Network backbone	ResNet101, Wav2Vec2, GloVe / MMIM
	Optimizer	Adam
	Learning rate	1×10^{-4}
	Weight decay	1×10^{-5} (MELD), 1×10^{-4} (MOSI)
	Number of training epochs	50 (MELD), 40 (MOSI)
IEMF	Inverse effectiveness coefficient Y	1.0
	Gating function $K(\cdot)$	$\tanh(\cdot)$

Table S2.

Comparison of the proposed method (w/ IEMF) and the vanilla baseline on ANN across three multimodal datasets—CRMEA-D, Kinetics-Sounds, and UrbanSound8K-AV—under four experimental settings (Normal, MSLR, OGM_GE, and LFM). Bold values indicate the highest accuracy achieved in each configuration.

Methods	CRMEA-D				Kinetics-Sounds				UrbanSound8K-AV			
	Normal	MSLR	OGM_GE	LFM	Normal	MSLR	OGM_GE	LFM	Normal	MSLR	OGM_GE	LFM
Vanilla	62.63	64.11	68.68	**64.11**	51.58	51.89	57.63	55.28	**97.90**	97.79	97.60	98.05
w/ IEMF	**63.44**	**65.59**	**71.10**	63.98	**56.17**	**55.86**	**64.61**	**63.15**	97.86	**97.98**	**99.24**	**98.63**

Table S3.

Experiments beyond Audio Visual. Visual and Language Fusion on Hateful Memes dataset. Comparison of the proposed method (w/ IEMF) and the vanilla baseline on three multimodal architectures—Concat BERT, MMBT-Grid, and ViLBERT—in terms of Accuracy (Acc.), Area Under the Receiver Operating Characteristic Curve (AUROC) and Macro F1 score (F1 (Macro). Bold values indicate the highest performance achieved in each configuration.

Methods	Hateful Memes dataset<br>Concat BERT			MMBT-Grid			ViLBERT		
	Acc.	AUROC	F1(Macro)	Acc.	AUROC	F1(Macro)	Acc.	AUROC	F1(Macro)
Vanilla	59.80	66.19	58.87	60.20	63.84	59.33	58.40	62.74	54.97
w/IEMF	**60.80**	**67.12**	**59.82**	**62.20**	**66.09**	**59.85**	**60.00**	**63.59**	**59.86**

Table S4.

Experiments on Tri-modal (Text-Audio-Visual) Fusion. Performance comparison on the MELD (emotion recognition) and CMU-MOSI (sentiment analysis) datasets. For MELD, the table reports F1 scores for individual emotion categories alongside the overall Weighted-F1. For CMU-MOSI, Binary Accuracy (Acc-2) and F1 scores are presented. Note that for these metrics, the values separated by a slash denote results for non-negative/negative (left) and positive/negative (right) classification tasks, respectively.

Methods	MELD								CMU-MOSI	
	anger	disgust	fear	joy	neutral	sadness	surprise	Weighted-F1	Acc-2	F1
Vanilla	68.75	41.91	4.26	15.25	42.03	2.22	**32.85**	49.77	81.63/82.93	81.64/82.99
w/ IEMF	**71.52**	**43.54**	**8.82**	**19.88**	**44.86**	**7.92**	30.72	**52.03**	**82.51**/**84.15**	**82.38**/**84.07**

Table S5.

Performance comparison on three audio-visual continual learning benchmarks (AVE-CI, K-S-CI, and VS100-CI) in terms of Mean Accuracy (↑) and Average Forgetting (↓). We evaluate three baseline methods (LwF, SSIL, and AV-CIL) and their variants augmented with the proposed IEMF module. Bold values indicate the best performance in each metric.

Method	AVE-CI		K-S-CI		VS100-CI	
	Mean Accuracy ↑	Avg Forgetting ↓	Mean Accuracy ↑	Avg Forgetting ↓	Mean Accuracy ↑	Avg Forgetting ↓
LwF	54.06	26.77	59.89	**15.26**	59.06	**17.91**
LwF w/ IEMF	**56.00**	**24.37**	**62.79**	17.25	**60.72**	18.25
SSIL	56.19	7.61	63.31	**4.66**	68.21	**8.53**
SSIL w/ IEMF	**58.12**	**6.25**	**65.18**	5.45	**70.01**	9.30
AV-CIL	61.86	24.31	70.63	11.03	70.15	**8.98**
AV-CIL w/ IEMF	**61.94**	**20.87**	**72.49**	**10.44**	**70.76**	9.49

Table S6.

Comparison results with different AVQA methods on the MUSIC-AVQA dataset, where different types of questions (audio-only, visual-only, and audio-visual) are evaluated.

Method	Audio Question (%)			Visual Question (%)			Audio-Visual Question (%)						Overall Avg. (%)
	Counting	Comparative	Avg	Counting	Location	Avg	Existential	Location	Counting	Comparative	Temporal	Avg	
Baseline	77.20	62.06	71.60	74.15	76.79	75.48	81.71	67.43	**62.57**	61.61	62.99	67.34	70.24
Baseline w/ IEMF	**77.40**	**63.89**	**72.40**	**75.23**	**76.79**	**76.02**	**82.11**	**69.07**	59.55	**62.87**	**64.93**	**67.87**	**70.82**
ST-AVQA	77.59	62.23	71.90	73.89	75.57	74.74	**82.81**	68.45	**63.00**	60.45	62.86	67.61	70.26
ST-AVQA w/ IEMF	**79.84**	**65.39**	**74.49**	**74.65**	**76.63**	**75.65**	82.11	**69.47**	62.68	**62.51**	**64.20**	**68.33**	**71.36**

Table S7.

Generalizability analysis across diverse network architectures on three benchmarks. We report the top-1 accuracy (%) on Spiking Neural Networks (SNN) with ResNet and Transformer backbones. ``Vanilla" denotes the baseline fusion method, and ``w/ IEMF" denotes the method augmented by our proposed method.

Architecture	Fusion Method	CREMA-D		Kinetics-Sounds		UrbanSound8K-AV	
		Vanilla	w/ IEMF	Vanilla	w/ IEMF	Vanilla	w/ IEMF
ResNet-18 (SNN)	Normal	63.04	**64.65**	53.12	**54.97**	**98.02**	97.79
	MSLR	63.70	**64.38**	53.19	**56.63**	97.98	**98.05**
	OGM_GE	69.49	69.35	57.28	**62.88**	97.67	**99.35**
	LFM	63.58	**64.78**	54.63	**63.53**	98.13	**98.63**
Transformer (SNN)	SCA Cross-Attn	68.55	**69.09**	56.86	**58.17**	97.21	**97.25**
Transformer (SNN)	Spatial Cross-Attn	71.10	**73.11**	57.52	**58.83**	**97.63**	97.48
Transformer (SNN)	SpatialTemporal Cross-Attn	71.77	**72.98**	57.48	**59.02**	**97.63**	97.48

Table S8.

Comparison of the proposed method (w/ IEMF) and the other gating methods.

Methods	CRMEA-D				Kinetics-Sounds				UrbanSound8K-AV			
	Normal	MSLR	OGM_GE	LFM	Normal	MSLR	OGM_GE	LFM	Normal	MSLR	OGM_GE	LFM
Shannon Information Gating	62.63	64.11	68.68	**64.11**	51.58	51.89	57.63	55.28	**97.90**	97.79	97.60	98.05
MOE Gating	61.96	63.84	69.09	63.44	51.54	51.70	57.09	54.78	97.86	97.82	97.36	97.60
IEMF	**63.44**	**65.59**	**71.10**	63.98	**56.17**	**55.86**	**64.61**	**63.15**	97.86	**97.98**	**99.24**	**98.63**

Table S9.

Performance comparison of the ANN model on CREMA-D: Vanilla vs. IEMF across varying training data ratios ($20\% - 100\%$) under Normal and OGM_GE settings.

Methods	Normal					OGM_GE				
	0.2	0.4	0.6	0.8	1.0	0.2	0.4	0.6	0.8	1.0
Vanilla	50.00	**56.85**	59.95	60.62	62.63	50.94	58.33	59.14	64.92	68.68
w/ IEMF	**53.09**	56.72	**60.75**	**62.07**	**63.44**	**53.50**	**59.95**	**64.11**	**66.39**	**71.10**

Table S10.

Complexity and performance analysis. The subscripts denote the change relative to the Vanilla baseline (red indicates performance gain, gray indicates computational cost).

Method	Params	Memory	FLOPs	Training Time	Inference Time	Accuracy
Vanilla	22.374943 M	6802 MB	21.811443 G	2.85 h	9.74 s	51.58%
Ours	$22.406749\text{M}_{(+0.03)}$	$6814\text{MB}_{(+12)}$	$21.811539\text{G}_{(\approx 0)}$	$2.96\ \text{h}_{(+0.11)}$	$9.74\ \text{s}_{(+0.00)}$	$\mathbf{56.17}_{(+\mathbf{4.59})}$

Table S11.

Statistical analysis of performance on CREMA-D across five random seeds. The table presents results for individual runs under the Normal setting, alongside summary statistics (mean ± standard deviation). P-values denote statistical significance derived from paired t-tests comparing IEMF against the Vanilla baseline.

Methods	Random Seeds					Statistics	
	0	42	2025	3917	8665	Average	p-value
Vanilla	97.67	97.56	97.90	97.63	97.90	97.73 ± 0.16	-
w/ IEMF $\gamma = 0.5$	98.05	97.82	98.02	98.02	98.21	$\mathbf{98.02 \pm 0.14}$	0.004
w/ IEMF $\gamma = 1.0$	97.86	97.67	97.86	97.86	98.09	97.87 ± 0.15	0.048
